# Transferrin ameliorates retinal degeneration by mediating the dimerization of all-*trans*-retinal

**DOI:** 10.1016/j.jbc.2024.108054

**Published:** 2024-12-09

**Authors:** Lei Tao, Danxue He, Yuling Chen, Kunhuan Yang, Beiting He, Peixin Cai, Binxiang Cai, Chunyan Liao, Zuguo Liu, Shiying Li, Jingmeng Chen, Yalin Wu

**Affiliations:** 1 Department of Ophthalmology, The First Affiliated Hospital of Xiamen University, School of Medicine, Xiamen University, Xiamen, Fujian, China; 2 Fujian Provincial Key Laboratory of Ophthalmology and Visual Science, Fujian Engineering and Research Center of Eye Regenerative Medicine, Eye Institute of Xiamen University, School of Medicine, Xiamen University, Xiamen, Fujian, China; 3 School of Medicine, Xiamen University, Xiamen, Fujian, China; 4 Shenzhen Research Institute of Xiamen University, Shenzhen, Guangdong, China

**Keywords:** transferrin, all-*trans*-retinal, dimerization, macular degeneration, retinal function

## Abstract

High levels of all-*trans*-retinal (atRAL) in the retina is considered to be responsible for the development of autosomal recessive Stargardt’s disease (STGD1) and dry age-related macular degeneration (dAMD). Two bisretinoids, all-*trans*-retinal dimer (atRAL-dimer) and *N*-retinyl-*N*-retinylidene ethanolamine (A2E), form from the dimerization of atRAL in the retina but they possess much lower toxicity and phototoxicity toward retinal pigment epithelium (RPE) cells than atRAL. Here, we introduced a novel function of transferrin (TRF) in mediating the conversion of atRAL into atRAL-dimer and A2E, which effectively protected the retina from damage by atRAL and prevented retinal function decline in mice, and rescued atRAL-loaded RPE cells. Moreover, TRF-mediated conversion of atRAL to atRAL-dimer and A2E required the help of bicarbonate ions (HCO_3_^–^). atRAL had the capacity to stimulate the expression of TRF in RPE and photoreceptor cells as well as RPE/choroid and neural retina of mice, reflecting that the elevation of TRF levels by atRAL is most likely to help defy level increase and cytotoxicity of atRAL through facilitating its dimerization and thereby serves as a mechanism of retinal self-protection. Our findings offer a promising avenue for the treatment of retinopathies characterized by disrupted clearance of atRAL.

The responsibility of retina-specific ABC transporter 4 (ABCA4) and all-*trans*-retinol (atROL) dehydrogenase 8 (RDH8) in the visual (retinoid) cycle is to eliminate free all-*trans*-retinal (atRAL) (1, 2, 3). It has been acknowledged that mutations in the *Abca4* gene cause autosomal recessive Stargardt’s disease (STGD1), a monogenic form of hereditary macular dystrophy (4, 5), and they increase the risk for dry age-related macular degeneration (dAMD) (6, 7, 8). *Rdh8* is considered as an assistant susceptibility gene for STGD1 and dAMD, and its knockout in mice does not incite the degeneration of retina but slows the reduction of atRAL to atROL in the visual (retinoid) cycle and accelerates retinal atrophy in *Abca4* mutant mice (9, 10). Mice carrying a double knockout of *Abca4* and *Rdh8* genes (*Abca4*^*−/−*^*Rdh8*^*−/−*^ mice) show features of STGD1 and dAMD in humans as well as rapid accumulation of atRAL condensation products and severe defects in clearance of atRAL in the retina (10, 11). Moreover, exposure of *Abca4*^*−/−*^*Rdh8*^*−/−*^ mice to light rapidly enhances atRAL levels in the retina and accelerates damage to the retina (12, 13).

A large body of evidence demonstrate that aberrantly high levels of atRAL in neural retina and the RPE results from disrupted visual (retinoid) cycle and are closely associated with STGD1 and dAMD resulting in irreversible blindness in humans, for which there are so far no cures (10, 11, 12, 13, 14, 15, 16, 17, 18, 19, 20, 21). All-*trans*-retinal dimer (atRAL-dimer) and *N*-retinylidene-*N*-retinylethanolamine (A2E) (Fig. S1) are two bisretinoids derived from condensation reactions of atRAL in the retina (22, 23, 24). Several lines of investigation indicate that either atRAL-dimer or A2E is much less toxic and phototoxic to RPE cells than their precursor atRAL, suggesting that endogenous production of atRAL-dimer and A2E is likely a protective mechanism to prevent damage to the retina by the free form of atRAL (25, 26, 27). Accordingly, the conversion of atRAL into atRAL-dimer and A2E by a safe compound may be a feasible approach for the therapy of STGD1 and dAMD.

Transferrin (TRF) is an iron transporter and its primary function is to accept iron from plasma and to deliver iron into cells *via* TRF receptor 1 (TRFr1) and clathrin-mediated endocytosis (28, 29). To our knowledge, TRF is not only highly abundant in human neural retina and RPE (30, 31) but also it is significantly upregulated in the retina of patients with dAMD (32). However, the relationship of TRF to macular degeneration has yet to be clarified. In this communication, our aim is to elucidate the role of TRF in the dimerization of atRAL and treatment of STGD1 and dAMD.

## Results

### TRF converts atRAL into atRAL-dimer in a cell-free system

First, it is necessary to briefly explain how we find the ability of apo-transferrin (apo-TRF) to mediate the dimerization of atRAL. When atRAL is incubated overnight in Dulbecco's Modified Eagle Medium (DMEM) supplemented with 10% fetal bovine serum, high performance liquid chromatography (HPLC) analysis of the mixtures unexpectedly reveals the production of atRAL-dimer. Since all components of DMEM with serum are well known, we further analyze the effect of each component on the conversion of atRAL into atRAL-dimer, whereas they fail to dimerize atRAL alone. After careful consideration, the combined effect of two or more components in DMEM with serum on the dimerization of atRAL is examined. TRF and bicarbonate ions (HCO_3_^–^) are two components of DMEM with serum. It is observed that apo-TRF and HCO_3_^–^ work together to convert atRAL into atRAL-dimer.

**Figure 1:**
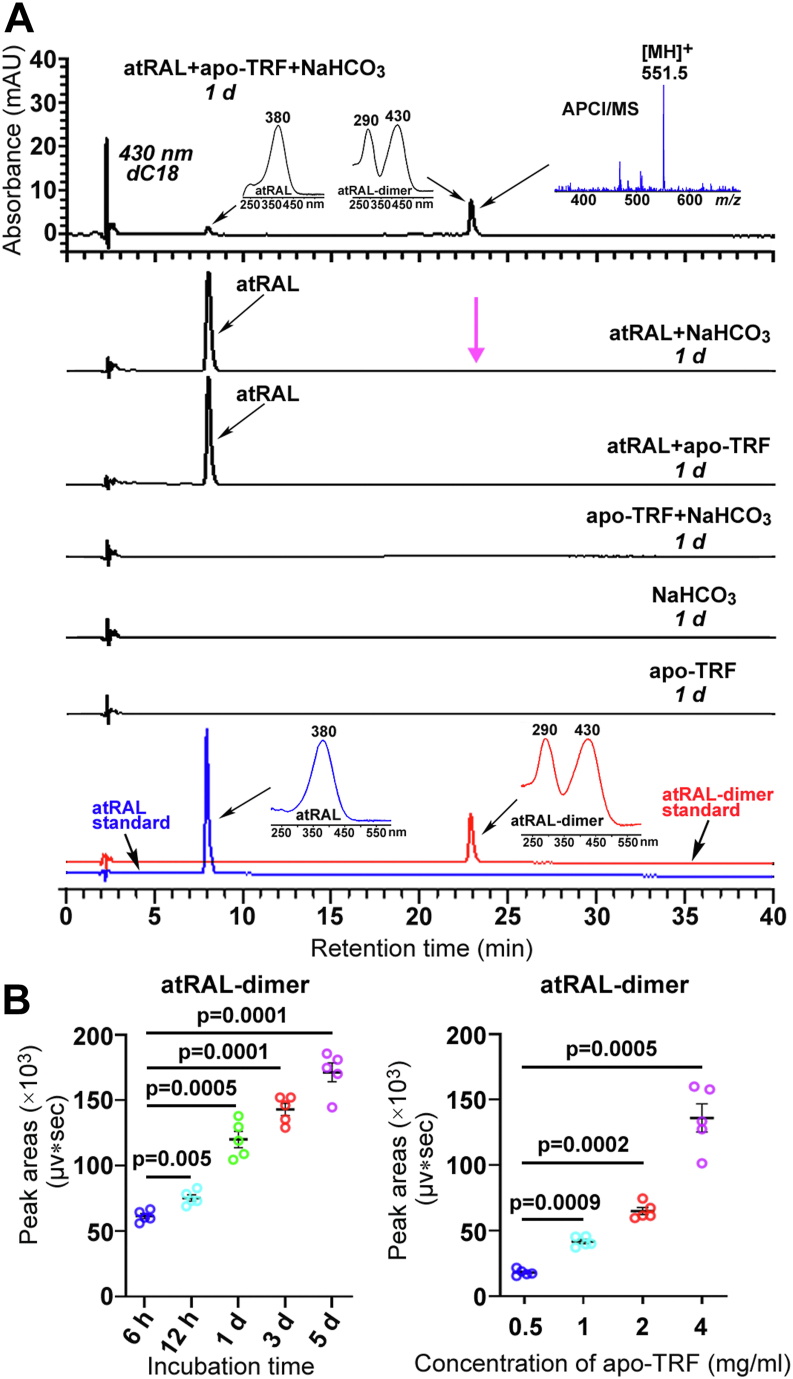
**apo-TRF mediates formation of atRAL-dimer from atRAL in the cell-free system.***A,* typical HPLC chromatograms (dC18 column; 430 nm monitoring) generated from reaction mixtures of atRAL (40 μM), apo-TRF (4 mg/ml) and NaHCO_3_ (24 mM), atRAL and NaHCO_3_, atRAL and apo-TRF, or apo-TRF and NaHCO_3_ as well as NaHCO_3_ or apo-TRF alone; incubations at 37 ^o^C in the dark for 1 day in 500 μl water. Right inset, corroboration of atRAL-dimer by a method of UPLC/APCI-MS. Mass spectrum of the atRAL-dimer peak from the 1-day reaction of atRAL, apo-TRF and NaHCO_3_ with the APCI source operated in positive ion mode. atRAL-dimer has a mass of 550.86. Also shown were HPLC profiles of atRAL and atRAL-dimer standards. *Insets* on the *left* or *right*, UV-visible absorbance spectra of atRAL and atRAL-dimer. *B,* the levels of atRAL-dimer, 6 h, 12 h, 1 day, 3 days and 5 days after treatment of 4 mg/ml apo-TRF with 40 μM atRAL and 24 mM NaHCO_3_ in 500 μl water at 37^o^C in the dark were measured, or they, 6 h after incubation of 40 μM atRAL with 0.5, 1, two and 4 mg/ml apo-TRF in 500 μl water containing 24 mM NaHCO_3_ at 37 ^o^C in the dark, were quantified. Each value represents mean ± SD (n = 5). One-way ANOVA with Tukey’s post-test was performed for statistical analyses. d, day (s).

Next, we investigated the effect of more than 20 endogenous proteins on the atRAL dimerization, and found that only apo-TRF had the capacity to convert atRAL into atRAL-dimer in the presence of sodium bicarbonate (NaHCO_3_) (Table S1 and Fig. 1). Upon examination by HPLC, 1-day incubation of 40 μM atRAL, 4 mg/ml apo-TRF and 24 mM NaHCO_3_ in 500 μl water at 37 ^o^C in the dark significantly induced the production of atRAL-dimer having a retention time of 22.9 min and absorbance maxima at 430 and 290 nm, but it was absent in the mixtures from reactions of atRAL with apo-TRF or NaHCO_3_ (Fig. 1*A*). Ultraperformance liquid chromatography/atmospheric pressure chemical ionization-mass spectrometry (UPLC/APCI-MS) analysis of the 430/290 nm adduct in positive ion mode disclosed an *m/z* peak at 551.5 (MH^+^) which was compatible with that of atRAL-dimer (theoretically, *m/z* 551.4, MH^+^) (Fig. 1*A*). Moreover, retention time, absorbance maxima and APCI-MS/MS spectrum of the 430/290 nm compound of interest were identical to those of synthetic atRAL-dimer (Fig. 1*A* and Fig. S2). The data manifested that the 430/290 nm product in reaction mixtures of atRAL, apo-TRF and NaHCO_3_ was atRAL-dimer. Quantification by measuring peak areas showed that the amounts of atRAL-dimer in reaction mixtures of 40 μM atRAL, 4 mg/ml apo-TRF and 24 mM NaHCO_3_ were elevated with the increasing of incubation time (Fig. 1*B*). As expected, when the mixtures from 1-day reactions of 40 μM atRAL and 24 mM NaHCO_3_ with apo-TRF were assessed by reverse-phase HPLC, the levels of atRAL-dimer were increased with the increase of apo-TRF concentrations (Fig. 1*B*). Taken together, these lines of evidence reveal that apo-TRF facilitates the conversion of atRAL into atRAL-dimer with the help of HCO_3_^–^.

### TRF aggravates the formation of DP-A2PE from reactions of atRAL with DP-PE in a cell-free system

**Figure 2:**
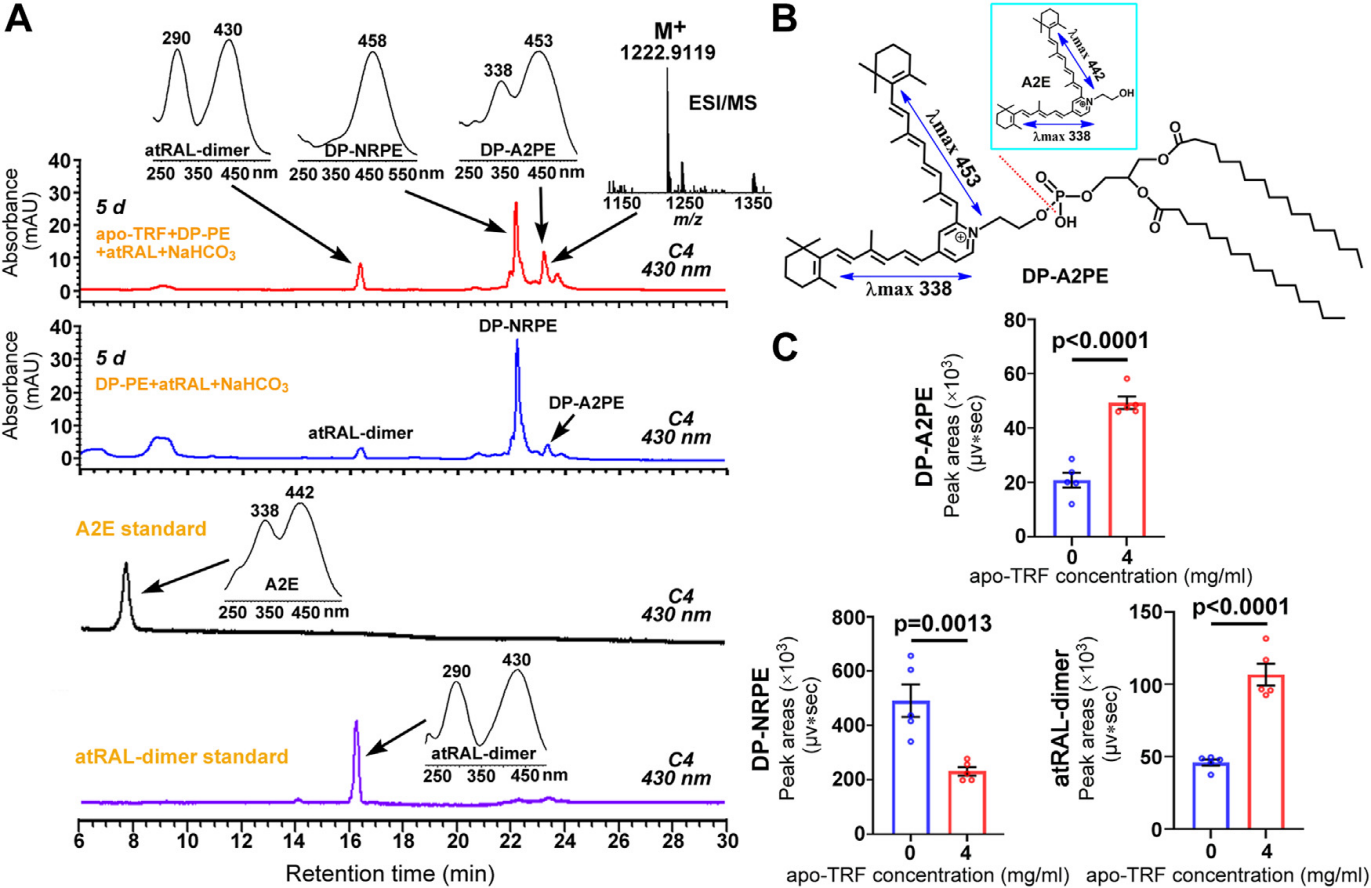
**apo-TRF stimulates formation of DP-A2PE, the precursor of A2E, from atRAL and DP-PE in the cell-free system.***A,* representative HPLC profiles (C4 column; 430 nm monitoring) generated from reaction mixtures of 40 μM atRAL, 4 mg/ml apo-TRF, 800 μM DP-PE and 24 mM NaHCO_3_, or atRAL, DP-PE and NaHCO_3_; incubations at 37 ^o^C in the dark for 5 days in 500 μl water. *Inset* on the *right*, corroboration of DP-A2PE by a method of HPLC/ESI-HRMS. Mass spectrum of the DP-A2PE peak from the 5-days reaction of atRAL, apo-TRF, DP-PE and NaHCO_3_ with the ESI source operated in positive ion mode. DP-A2PE has a mass of 1222.9143. *Left insets*, UV-visible absorbance spectra of atRAL-dimer, DP-NRPE and DP-A2PE. *B,* structures, UV-visible absorbance maxima (nanometers) and electronic transition assignments (↔) of DP-A2PE and A2E. Phosphate hydrolysis (dashed lines) of DP-A2PE gives rise to A2E. Also shown were HPLC chromatograms of A2E and atRAL-dimer standards. *Insets* on the *left* or *right*, UV-visible absorbance spectra of A2E and atRAL-dimer. *C,* quantification of DP-A2PE, DP-NRPE and atRAL-dimer. atRAL (40 μM) was incubated for 5 days with apo-TRF (0 or 4 mg/ml), 800 μM DP-PE and 24 mM NaHCO_3_ in 500 μl water at 37 ^o^C in the dark. Each value represents mean ± SD (n = 5). Statistical analyses were conducted by Student’s *t* test. d, days.

Studies in the past have documented that the biogenesis of A2E involves the formation of the precursor, *N*-retinylidene-*N*-retinylphosphatidylethanolamine (A2PE), and the intermediate, *N*-retinyl-phosphatidylethanolamine (NRPE) (33). It should be noted that A2PE and NRPE in the retina are generated from complex phosphatidylethanolamine (PE) consisting of a mixture of fatty acids with varying lengths and degrees of unsaturation (33). Here, dipalmitoyl-L-α-phosphatidylethanolamine (DP-PE) was employed to yield DP-A2PE and DP-NRPE as references A2PE and NRPE, respectively. There is already evidence that DP-A2PE absorbs light at maximum wavelengths of 337 and 456 nm, and DP-NRPE absorbs light at a maximum wavelength of 456 nm (33). Analysis of reaction mixtures by HPLC manifested that 40 μM atRAL was reacted with 800 μM DP-PE for 5 days in the dark at 37 ^o^C in 500 μl water containing 24 mM NaHCO_3_ to produce DP-A2PE exhibiting absorbance maxima of 338 and 453 nm, as well as DP-NRPE with a maximum absorbance at the wavelength of 458 nm (Fig. 2*A*). Subjecting of the 338/453 nm compound to HPLC/electrospray ionization-high resolution mass spectrometry (HPLC/ESI-HRMS) in positive ion mode gave rise to an *m/z* peak at 1222.9119 (M^+^) which was identical to that of DP-A2PE (theoretically, *m/z* 1222.9143, M^+^) (Fig. 2*A*, inset). The addition of 4 mg/ml apo-TRF visibly facilitated the formation of DP-A2PE and atRAL-dimer but reduced the levels of DP-NRPE in reactions of atRAL with DP-PE in water containing NaHCO_3_ (Fig. 2, *A–C*). Since phosphate hydrolysis of DP-A2PE is required to produce A2E (Fig. 2*B*), A2E was absent in reactions of atRAL and DP-PE with or without apo-TRF in water containing NaHCO_3_ (Fig. 2*A*). These data suggest that, with the aid of HCO_3_^–^, apo-TRF is capable of increasing the production of A2PE, the direct precursor of A2E, from reactions of atRAL with PE.

### TRF expression is elevated by atRAL in RPE and photoreceptor cells

**Figure 3:**
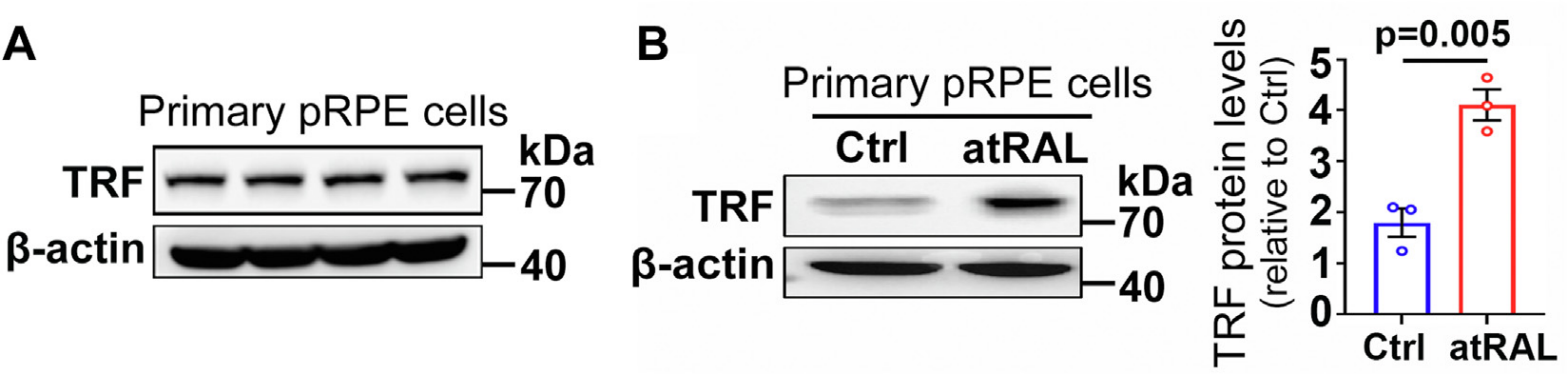
**The expression of TRF is evident and it is significantly increased by atRAL in primary pRPE cells.***A,* Western blots of TRF with a molecular weight of 78 kDa in primary pRPE cells. β-actin served as an internal control. Molecular-weight markers (kDa) are indicated on the *right* side of the blots. *B,* immunoblots of TRF in primary pRPE cells exposed for 6 h to 40 μM atRAL or DMSO alone. Protein levels of TRF were normalized to those of β-actin and expressed as fold changes compared with DMSO-treated controls. Statistical analyses were performed using Student’s *t* test.

We first confirmed that TRF was clearly expressed in primary porcine RPE (pRPE) cells, human RPE cell line ARPE-19 and murine photoreceptor cell line 661W (Fig. 3*A*, and Fig. S3, *A* and *C*). Western blotting was used to detect the expression of TRF in primary pRPE cells incubated for 6 h with 40 μM atRAL, ARPE-19 cells exposed for 6 and 12 h to 15 μM atRAL, and 661W photoreceptor cells treated for 6 h with 5 μM atRAL. The results showed that protein levels of TRF were significantly or time-dependently increased by atRAL in RPE and photoreceptor cells (Fig. 3*B*, and Fig. S3, *B* and *D*).

### TRF rescues atRAL-loaded RPE cells by promoting the conversion of atRAL into atRAL-dimer and A2E

**Figure 4:**
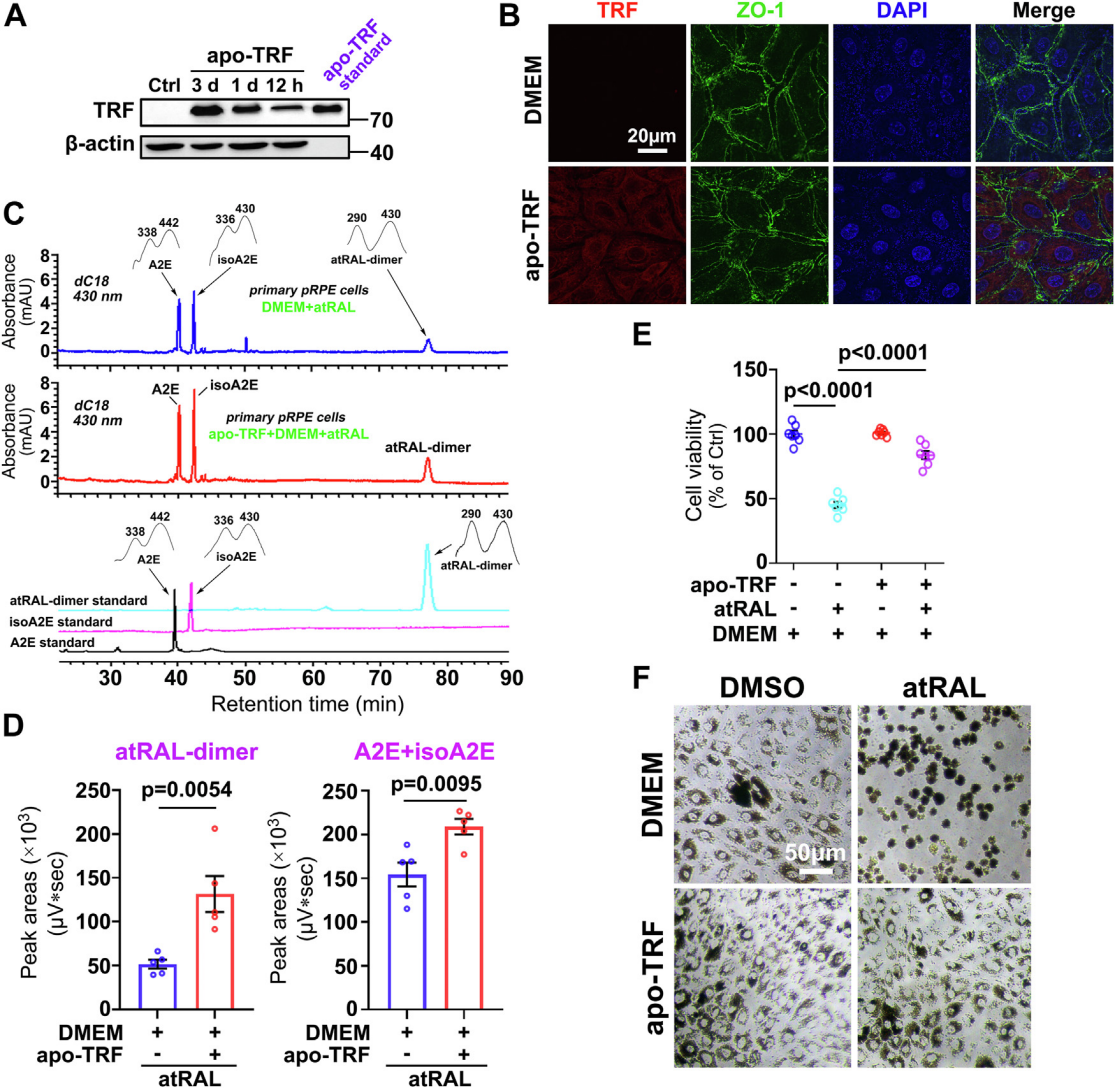
**Treatment with apo-TRF reduces the cytotoxicity of atRAL in primary pRPE cells through promoting the dimerization of atRAL.***A,* Western blots of TRF in primary pRPE cells treated with or without 2 mg/ml apo-TRF for 12 h, 1 day and 3 days in DMEM. Commercial apo-TRF standard served as a positive control. *B,* internalization of apo-TRF was detected by co-staining with TRF (*red*), ZO-1 (*green*) and DAPI (*blue*) using confocal laser scanning fluorescence microscopy. Primary pRPE cells were incubated for 3 days with 2 mg/ml apo-TRF or DMEM alone. *Scale bars,* 20 μm. *C,* typical HPLC chromatograms (dC18 column; 430 nm monitoring) were generated with the extracts from the same number of primary pRPE cells that were pretreated with 2 mg/ml apo-TRF for 3 days and then exposed for 1 day to 40 μM atRAL in DMEM. Control cells were cultured in DMEM for 3 days, followed by 1 day of treatment with atRAL. Also shown were HPLC profiles of synthetic standards for A2E, isoA2E and atRAL-dimer. *Insets*, UV-visible absorbance spectra of A2E, isoA2E and atRAL-dimer. *D,* the levels of atRAL-dimer and A2E were quantified using Empower version 3 software. The amounts of A2E were calculated as the sum of the levels of A2E and isoA2E. Each value represents mean ± SD (n = 5). mAU, milliabsorbance unit. Student’s *t* test was conducted for statistical analyses. *E* and *F,* cell viability and cellular morphology were assessed by MTS assay and light microscopy, respectively. Primary pRPE cells were pretreated for 3 days with 2 mg/ml apo-TRF in DMEM, followed by 1-day incubation with or without 40 μM atRAL. Cells treated with atRAL or DMSO alone in DMEM serve as controls. *Scale bars* in *F*, 50 μm. One-way ANOVA with Tukey’s post-test in *E* was conducted for statistical analyses. d, day (s).

Apo-TRF was incubated for 12 h, 1 day and 3 days with primary pRPE cells or ARPE-19 cells in DMEM without serum. The results of Western blotting showed that protein levels of TRF were significantly elevated in RPE cells and they were extremely significant on the third day (Fig. 4*A* and Fig. S4*A*). To further corroborate the internalization of apo-TRF, we performed immunofluorescence co-staining for TRF and ZO-1 in primary pRPE cells and ARPE-19 cells treated for 3 days with or without apo-TRF in serum-free DMEM. As expected, confocal imaging analysis revealed that the increase of TRF protein levels was clearly visualized within RPE cells (Fig. 4*B* and Fig. S4*B*). Furthermore, primary pRPE cells and ARPE-19 cells after exposure to apo-TRF were rinsed three times by phosphate buffer saline (PBS). Following the addition of fresh DMEM, Western blot analysis of the cell lysates and supernatant indicated that RPE cells carrying exogenous TRF were completely washed (Fig. S5).

Next, primary pRPE cells and ARPE-19 cells were incubated for 3 days with or without 2 mg/ml apo-TRF in serum-free DMEM, and then exposed for 1 day to 40 and 15 μM atRAL, respectively. IsoA2E is a C13C14 double-bond *Z*-isomer of A2E (Fig. S1) (23). It should be mentioned here that the amount of A2E is generally evaluated as the sum of levels of A2E and isoA2E due to the interconversion between them. Analysis of cellular extracts by HPLC disclosed that the conversion of atRAL into atRAL-dimer and A2E was further increased by exogenous addition of apo-TRF (Fig. 4, *C* and *D*, and Fig. S4, *C* and *D*). The results of CellTiter 96 AQueous One Solution Cell Proliferation assay (MTS) demonstrated that cell viability following exposure to atRAL was up by 38.57% in apo-TRF-treated primary pRPE cells and 59.1% in apo-TRF-exposed ARPE-19 cells (Fig. 4*E* and Fig. S4*E*). Notably, treatment with apo-TRF evidently reversed damage to the morphology of primary pRPE cells and ARPE-19 cells in response to atRAL (Fig. 4*F* and Fig. S4*F*). These findings imply that apo-TRF protects atRAL-loaded RPE cells by its ability to catalyze the dimerization of atRAL.

### TRF expression is enhanced in neural retina and RPE/choroid of light-exposed *Abca4*^*−/−*^*Rdh8*^*−/−*^ mice

**Figure 5:**
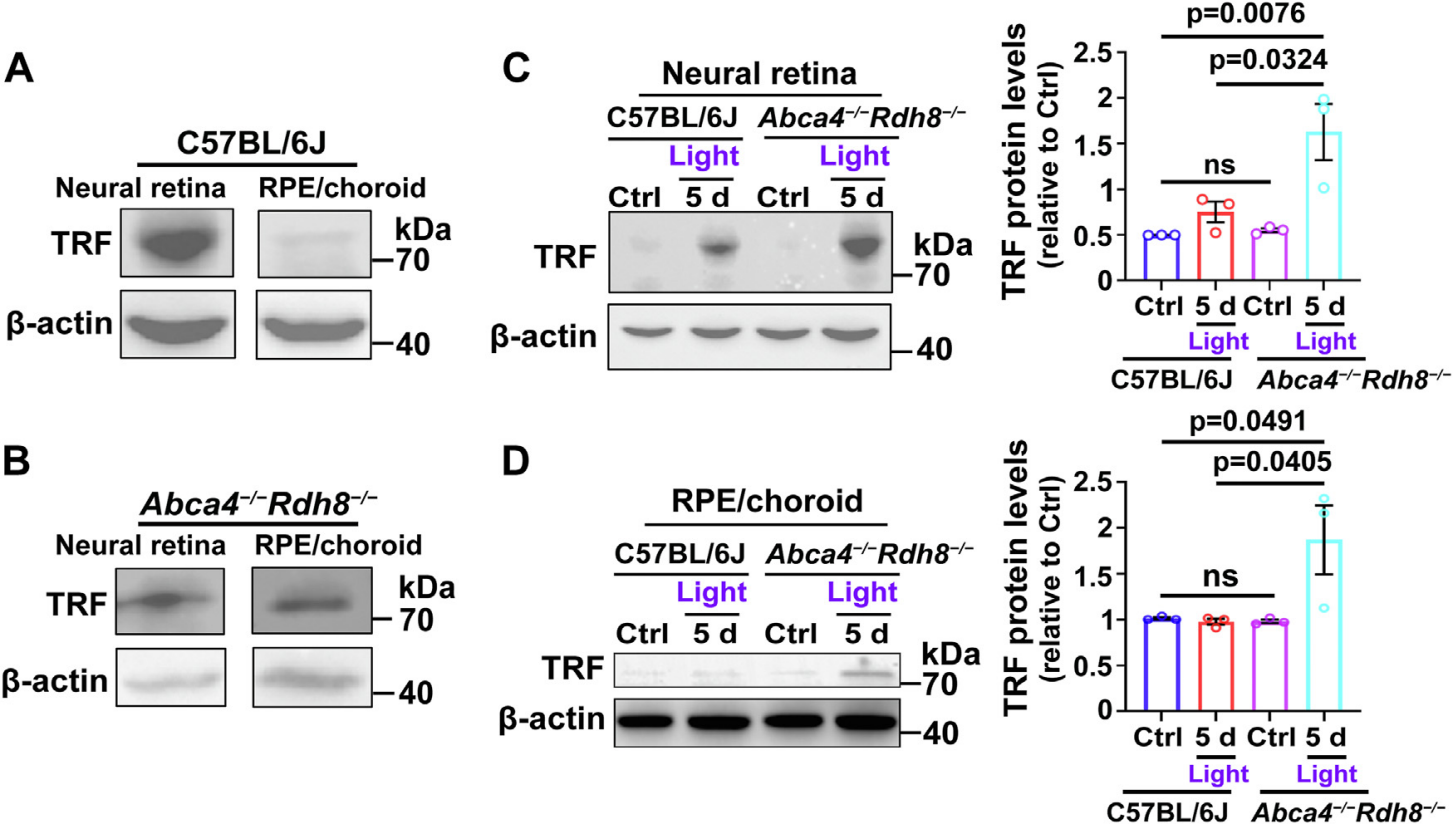
**Light exposure aggravates the expression of TRF in neural retina and RPE/choroid of *Abca4***^***−/−***^***Rdh8***^***−/−***^**mice.***A,* Western blots of TRF in neural retina and RPE/choroid from four-week-old C57BL/6J mice. *B,* immunoblots of TRF in neural retina and RPE/choroid from *Abca4*^*−/−*^*Rdh8*^*−/−*^ mice at 4 weeks of age. *C* and *D,* the pupils of 48-h dark-adapted C57BL/6J or *Abca4*^*−/−*^*Rdh8*^*−/−*^ mice aged 4 weeks were dilated with 1% tropicamide, followed by exposure of the mice to 10,000 lx LED light for 1 h. Eyeballs were harvested at day 5 upon light illumination and then dissected for neural retinas and RPE/choroids. Western blotting was used to examine the expression of TRF protein in neural retina and RPE/choroid, respectively. Protein levels of TRF were normalized to those of β-actin, and shown as fold changes relative to light-free C57BL/6J mice. Statistical analyses in *C* and *D* were carried out by one-way ANOVA with Tukey’s post-test.

Western blot analysis showed that the expression of TRF was present in neural retina and RPE/choroid of C57BL/6J wild-type and *Abca4*^*−/−*^*Rdh8*^*−/−*^ mice at 4 weeks of age (Fig. 5, *A* and *B*). Compared to four-week-old C57BL/6J mice, there were no obvious changes in protein levels of TRF in neural retina and RPE/choroid of age-matched *Abca4*^*−/−*^*Rdh8*^*−/−*^ mice and light-exposed C57BL/6J mice, whereas TRF expression was visibly elevated in neural retina and RPE/choroid of light-exposed *Abca4*^*−/−*^*Rdh8*^*−/−*^ mice aged 4 weeks (Fig. 5, *C* and *D*). These results suggest that light exposure stimulates the expression of TRF in the retina of mice defective for atRAL clearance.

### The production of atRAL-dimer and A2E is increased by exogenous apo-TRF in posterior eyecups of light-exposed *Abca4*^*−/−*^*Rdh8*^*−/−*^ mice

**Figure 6:**
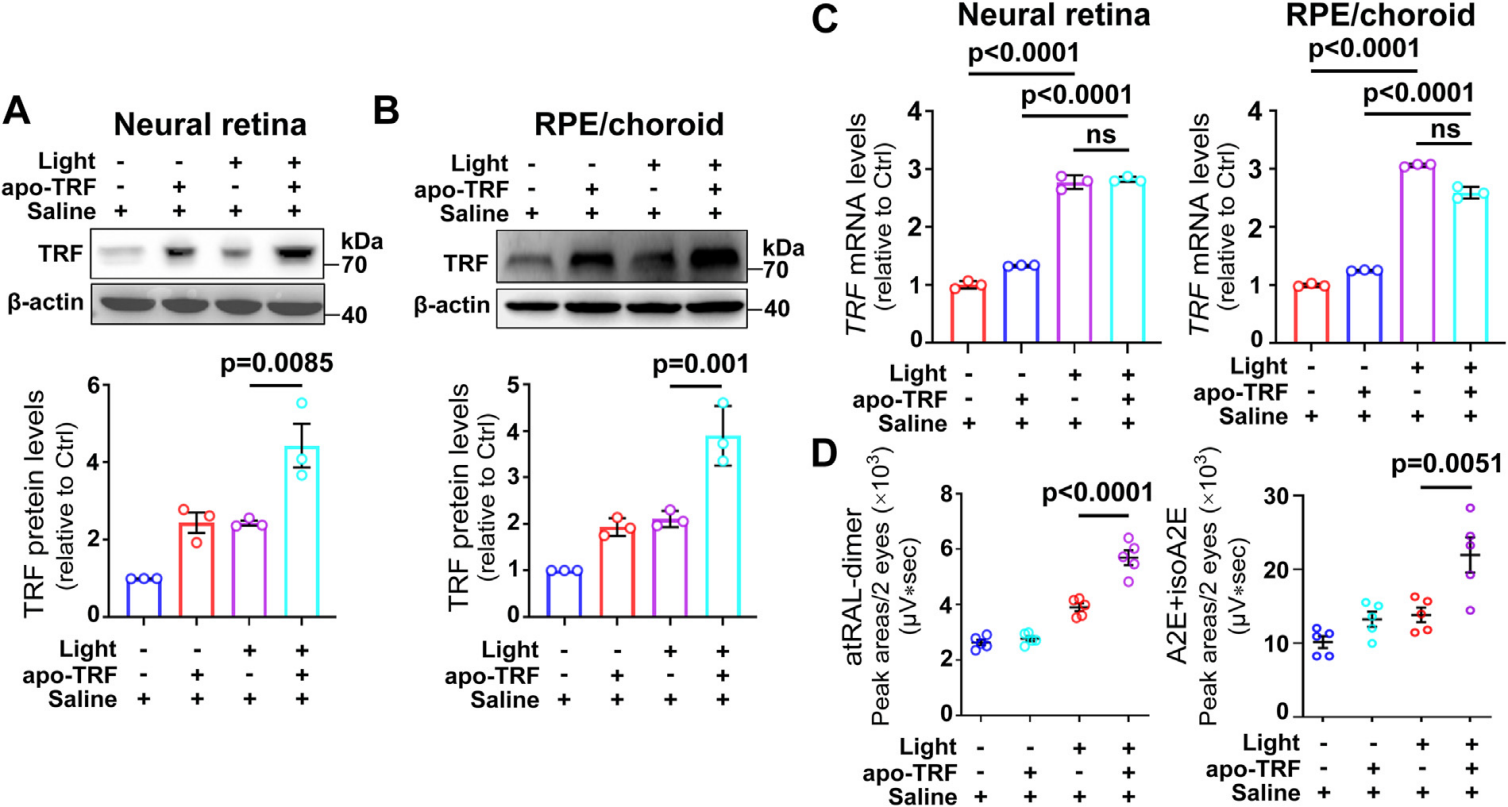
**Injection of exogenous apo-TRF into the vitreous of light-exposed *Abca4***^***−/−***^***Rdh8***^***−/−***^**mice increases protein levels of TRF in the retina and promotes the atRAL dimerization in posterior eyecups but it does not affect mRNA levels of *TRF* in the retina.** 48-h dark-adapted *Abca4*^*−/−*^*Rdh8*^*−/−*^ mice at 4 weeks of age were intravitreally administered with 2 μl apo-TRF solution (75 mg/ml in saline, 150 μg apo-TRF) or saline (vehicle). Four hours later, they were exposed for 1 h to 10,000 lx LED light and then kept in the dark for additional 4 days. Finally, eyeballs, 5 days after illumination, were collected and then dissected for neural retinas, RPE/choroids and posterior eyecups. Control *Abca4*^*−/−*^*Rdh8*^*−/−*^ mice were injected intravitreally with apo-TRF or saline without light exposure. *A* and *B,* Western blotting was employed to examine protein levels of TRF in extracts from each mouse neural retina (*A*) or RPE/choroid (*B*). Protein levels of TRF were normalized to those of β-actin and expressed as fold changes relative to saline-treated control *Abca4*^*−/−*^*Rdh8*^*−/−*^ mice. *C,* the qRT-PCR analysis of *TRF* gene in extracts from each mouse neural retina or RPE/choroid. The mRNA levels of *TRF* gene were shown as fold changes relative to saline-treated control *Abca4*^*−/−*^*Rdh8*^*−/−*^ mice. *D,* the levels of atRAL-dimer as well as the sum of A2E and isoA2E levels per two posterior eyecups were quantified using the HPLC system equipped with Empower version 3 software. Each value represents mean ± SD (n = 5). Statistical analyses in *A****–****D* were performed by one-way ANOVA with Tukey’s post-test.

To investigate the effect of exogenous apo-TRF on the dimerization of atRAL in the retina, 2 μl of 75 mg/ml apo-TRF (150 μg apo-TRF) in saline was intravitreally injected into light-exposed *Abca4*^*−/−*^*Rdh8*^*−/−*^ mice. The Western blotting and quantitative real-time polymerase chain reaction (qRT-PCR) results indicated that intravitreal administration of exogenous apo-TRF significantly increased protein levels of TRF in neural retina and the RPE/choroid of *Abca4*^*−/−*^*Rdh8*^*−/−*^ mice upon exposure to light but it had almost no effect on mRNA levels of *TRF* (Fig. 6, *A–C*), thereby suggesting that increased TRF in the retina of light-exposed *Abca4*^*−/−*^*Rdh8*^*−/−*^ mice after intravitreal injection of apo-TRF is exogenous. Moreover, exposure of *Abca4*^*−/−*^*Rdh8*^*−/−*^ mice receiving saline to light enhanced the expression of TRF at protein and mRNA levels in neural retina and the RPE/choroid (Fig. 6, *A–C*). Likewise, apo-TRF administration by intravitreal injection elevated protein levels of TRF in neural retina and the RPE/choroid of light-free *Abca4*^*−/−*^*Rdh8*^*−/−*^ mice but it did not affect mRNA levels of *TRF* (Figure 6, *A–C*). Analysis of posterior eyecup extracts by HPLC manifested that intravitreally administered apo-TRF visibly increased the levels of atRAL-dimer and A2E in posterior eyecups of *Abca4*^*−/−*^*Rdh8*^*−/−*^ mice in response to light (Fig. 6*D*). These data imply that intravitreally injected apo-TRF penetrates into the retina of mice with atRAL clearance defects where it helps promote the dimerization of atRAL.

### TRF effectively rescues retinal function and ameliorates retinal injury in *Abca4*^*−/−*^*Rdh8*^*−/−*^ mice with light exposure

**Figure 7:**
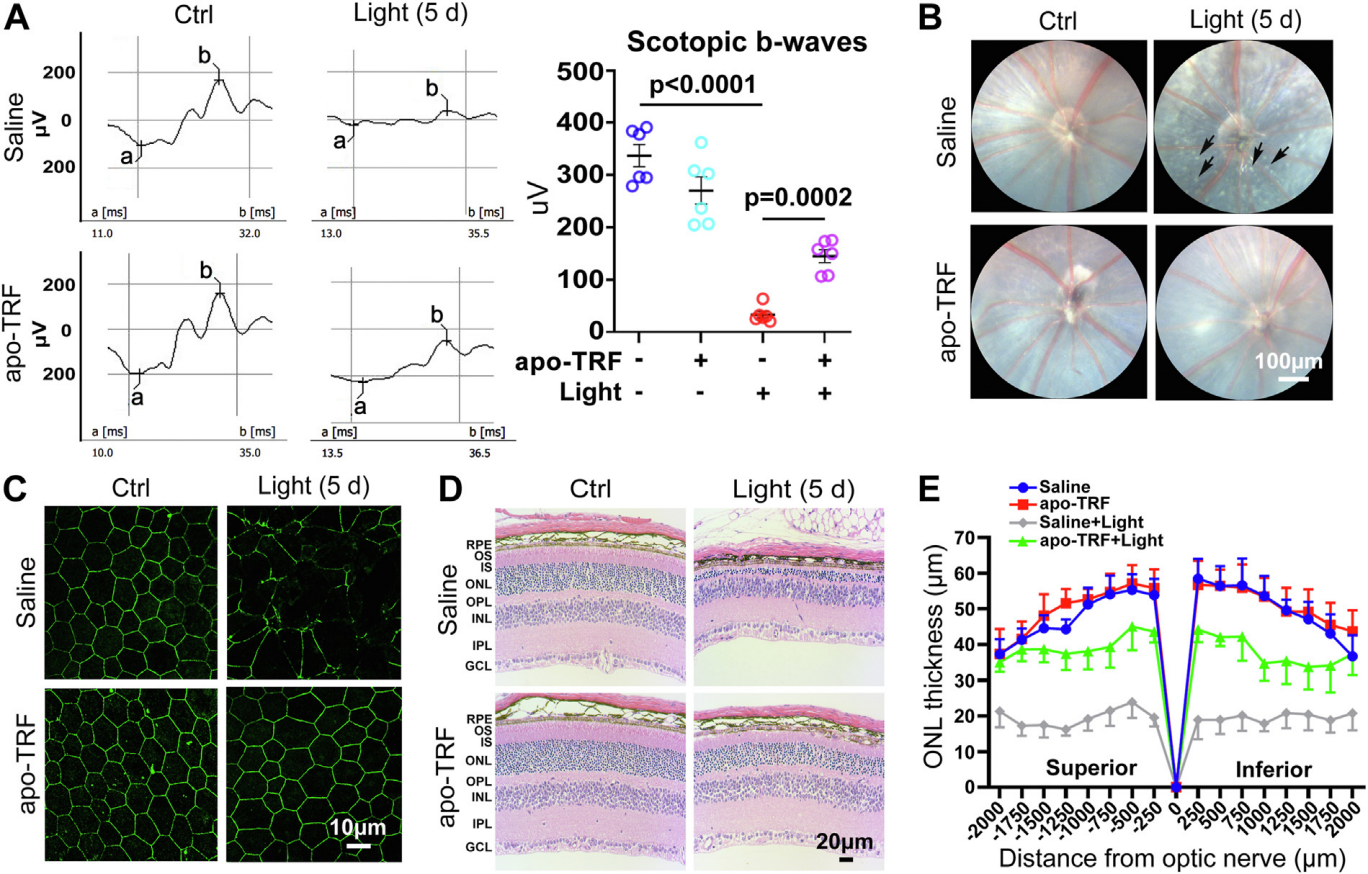
**Intravitreally injected apo-TRF effectively rescues retinal function and degeneration of light-exposed *Abca4***^***−/−***^***Rdh8***^***−/−***^**mice**. Four-week-old *Abca4*^*−/−*^*Rdh8*^*−/−*^ mice were dark adapted for 48 h, and then intravitreally injected with 2 μl apo-TRF solution (75 mg/ml in saline, 150 μg apo-TRF) or saline. Four hours later, the mice were illuminated with 10,000 lx LED light for 1 h after their pupils were dilated with 1% tropicamide. After being housed in the dark for additional 4 days, the resulting mice were used for the following studies. Control *Abca4*^*−/−*^*Rdh8*^*−/−*^ mice were administered intravitreally with apo-TRF or saline without exposure to light. Results in *A* to *E* were from at least six mice per group. *A,* retinal function of the mice was examined by scotopic ERG. Mice were stimulated with flashes of a light intensity of 10 cd s/m^2^. ERG amplitudes were quantified using a computer-based system. The amplitudes of b-waves were used to evaluate retinal function, and expressed as mean ± SD (n = 6). μV, microvolt. Statistical analyses were conducted by one-way ANOVA with Tukey’s post-test. *B,* the RPE morphology of each mouse was visualized by a small animal retinal imaging system (Optoprobe; OPIMG-L, UK). *Scale bars*, 100 μm. *C,* whole-mount ZO-1 immunofluorescence staining of the mouse RPE was visualized by confocal microscopy. *Scale bars*, 10 μm. *D,* histological analysis of each mouse neural retina was carried out using H&E staining. RPE, retinal pigment epithelium; OS, outer segment; IS, inner segment; ONL, outer nuclear layer; OPL, outer plexiform layer; INL, inner nuclear layer; IPL, inner plexiform layer; GCL, ganglion cell layer. *Scale bars,* 20 μm. *E,* ONL thickness measurement on the Superior-Inferior meridian (n = 8). Two-way ANOVA with Bonferroni's multiple comparison test. apo-TRF- *versus* saline-treated light-exposed *Abca4*^*−/−*^*Rdh8*^*−/−*^ mice (*p* < 0.001). Saline-treated light-exposed *versus* saline-treated control *Abca4*^*−/−*^*Rdh8*^*−/−*^ mice (*p* < 0.001).

Our previous reports have shown that compared to four-week-old C57BL/6J mice, retinal degeneration does not occur in age-matched *Abca4*^*−/−*^*Rdh8*^*−/−*^ mice and light-exposed C57BL/6J mice but it is observed in age-matched *Abca4*^*−/−*^*Rdh8*^*−/−*^ mice following exposure to light (11, 17). In this investigation, we further revealed that compared with C57BL/6J mice at 4 weeks of age, loss of retinal function did not appear in age-matched *Abca4*^*−/−*^*Rdh8*^*−/−*^ mice and light-exposed C57BL/6J mice yet it notably occurred in age-matched *Abca4*^*−/−*^*Rdh8*^*−/−*^ mice receiving light exposure, as evidenced by electroretinography (ERG) (Fig. S6). The data explained why *Abca4*^*−/−*^*Rdh8*^*−/−*^ mice at the age of 4 weeks were used in the present study. The scotopic ERG results demonstrated that the b-wave amplitudes were significantly reduced in *Abca4*^*−/−*^*Rdh8*^*−/−*^ mice after exposure to light but their decreases were dramatically prevented by intravitreal administration of 150 μg apo-TRF, suggesting the ability of intravitreally injected apo-TRF to rescue retinal function in *Abca4*^*−/−*^*Rdh8*^*−/−*^ mice receiving light exposure (Fig. 7*A*). *In vivo* retinal fundus imaging manifested that RPE degeneration in *Abca4*^*−/−*^*Rdh8*^*−/−*^ mice in response to light was visibly alleviated by intravitreal injection of apo-TRF (Fig. 7*B*). Moreover, whole-mount immunofluorescence staining of the RPE with an anti-ZO-1 antibody also verified that intravitreal apo-TRF administration remarkably maintained tight junctions in the RPE of *Abca4*^*−/−*^*Rdh8*^*−/−*^ mice upon exposure to light (Fig. 7*C*). Histological analysis of neural retina by hematoxylin and eosin (H&E) staining showed that intravitreally administered apo-TRF significantly attenuated the degeneration of neuroretinal photoreceptors and the reduction of the thickness of photoreceptor outer nuclear layer (ONL) in *Abca4*^*−/−*^*Rdh8*^*−/−*^ mice exposed to light (Figure 7, *D* and *E*). These lines of evidence imply that apo-TRF has the capacity to rescue retinal function and degeneration in mice featured by the overload of atRAL.

## Discussion

A great number of researches have indicated that high levels of atRAL in the retina contribute to the loss of photoreceptor and RPE cells in STGD1 and dAMD (10, 11, 12, 13, 14, 15, 16, 17, 18, 19, 20, 21). On the basis of the current understanding of the visual (retinoid) cycle (1), total atRAL should stay in the photoreceptor outer segment (POS), and wait for reduction of them into atROL. However, when timely clearance of atRAL is disturbed in the POS disks, the phagocytosis of shed POS disks by RPE cells explains why atRAL is also present in the RPE. Indeed, we have previously validated the existence of atRAL in the RPE/choroid of *Abca4*^*−/−*^*Rdh8*^*−/−*^ mice and humans by using HPLC (11).

In the retina, atRAL-dimer and A2E are produced from condensation reactions of atRAL (1). A2E is a relatively stable compound that can accumulate in RPE cells over time (34, 35, 36, 37, 38). Conversely, atRAL-dimer is observed at a very low level or even not detected in the RPE/choroid from humans, bovines and pigs as well as posterior eyecups of C57BL/6J mice, although the incubation of RPE cells or porcine rod outer segments with atRAL visibly leads to its formation (26, 27, 39, 40). A previous report from our laboratory has clarified that since atRAL-dimer shows higher susceptibility to light than A2E, light can rapidly break it into low-molecular-weight fragments which are likely to be metabolically eliminated (27), thereby explaining why atRAL-dimer, different from A2E, normally does not exhibit a time-dependent accumulation in RPE cells. Also, light exposure of atRAL-dimer gives rise to its photooxidation products, which may further undergo cleavage to yield low-molecular-weight fragments (27). Although we do not know whether the photooxidation of atRAL-dimer increases oxidative stress in RPE cells, exposure of atRAL-dimer-loaded RPE cells to light does not affect cell morphology and viability (27), thus revealing that photocleavage and photooxidation products of atRAL-dimer are likely to be nontoxic. We cannot exclude the possibility that atRAL-dimer accumulates beyond a critical level and thereby facilitates the death of RPE cells, but exposure of the retina to light in the life of human beings greatly reduces the chance of its excess accumulation (27, 40). By contrast, while A2E shows cytotoxicity and phototoxicity in RPE cells, the levels are significantly lower than those caused by atRAL (25, 40, 41, 42, 43, 44). Because atRAL contains an extremely active aldehyde group, it possesses a much stronger ability to kill RPE cells than atRAL-dimer and A2E (25, 26, 27). Indeed, similar to atRAL, atRAL-dimer and its photocleavage and photooxidation products have aldehyde groups. However, the activity of aldehyde groups in atRAL-dimer and its photocleavage and photooxidation products is possibly inactivated compared to that in atRAL, which explains why atRAL-dimer is much less toxic and phototoxic to RPE cells than atRAL. In addition to RPE cells, photoreceptor cells were also a site where TRF mediated the dimerization of atRAL (Figure 5, Figure 6, *A*, *C* and D, and Fig. S3, *C* and *D*). Therefore, the conversion of atRAL into atRAL-dimer and A2E not only decreases the levels of free atRAL but also ameliorates damage to photoreceptor and RPE cells in retinopathies arising from atRAL.

After screening more than two dozen endogenous proteins, only apo-TRF promoted the dimerization of atRAL with the aid of HCO_3_^–^ (Table S1). More importantly, uptake of exogenous apo-TRF into the retina effectively rescued retinal function and RPE/photoreceptor morphology from damage by atRAL.

We found that 40 μM atRAL was reacted with 800 μM DP-PE for 5 days in the dark at 37 ^o^C in 500 μl water containing 24 mM NaHCO_3_ to produce a small amount of atRAL-dimer (Fig. 2, *A* and *C*). A possible explanation for the phenomenon is that an atRAL/PE Schiff base conjugate may rearrange into atRAL-dimer. However, the 5-days incubation of 40 μM atRAL with 4 mg/ml apo-TRF alone in 500 μl water did not generate atRAL-dimer (Fig. S7), suggesting that the lysine side chain in apo-TRF, different from the primary amine of PE, is not responsible for converting atRAL into atRAL-dimer. Moreover, the addition of 4 mg/ml apo-TRF visibly facilitated the formation of atRAL-dimer in reactions of atRAL with DP-PE in water containing NaHCO_3_ (Figure 2, *A* and *C*), which further implies that different mechanisms involve apo-TRF-mediated the atRAL dimerization. We compared the impact of apo-TRF and holo-TRF on the conversion of atRAL into atRAL-dimer with the help of HCO_3_^–^. apo-TRF and holo-TRF at the concentration of 4 mg/ml were incubated for 1 day with 40 μM atRAL in 500 μl water containing 24 mM NaHCO_3_ at 37^o^C in the dark, respectively. HPLC analysis showed that apo-TRF better promoted the conversion of atRAL into atRAL-dimer than holo-TRF (Fig. S8), which in turn suggests the important role of iron in repressing TRF-mediated the atRAL dimerization.

HCO_3_^–^ is a major intracellular anion responsible for maintaining PH homeostasis in mammalian cells (45, 46). Studies in the past have revealed a function of HCO_3_^–^ in sustaining the activity of TRF to bind with metal ions (47). In our acellular assays, the addition of HCO_3_^–^ was a corequisite to apo-TRF mediated formation of atRAL-dimer from atRAL (Fig. 1*A* and Table S1). We also examined the effect of hydrogen phosphate ions (HPO_4_^2–^) or sulfate ions (SO_4_^2–^) on apo-TRF mediated conversion of atRAL into atRAL-dimer. Na_2_HPO_4_ and Na_2_SO_4_ at the concentration of 24 mM were incubated for 1 day with 4 mg/ml apo-TRF and 40 μM atRAL in 500 μl water at 37 ^o^C in the dark, respectively. HPLC analysis revealed that either HPO_4_^2–^ or SO_4_^2–^ failed to facilitate apo-TRF mediated conversion of atRAL into atRAL-dimer, thereby reflecting that HCO_3_^–^ plays a specific role in promoting apo-TRF mediated the atRAL dimerization.

However, in our cell-based experiments, the dimerization of atRAL occurred without adding exogenous apo-TRF and NaHCO_3_, and it depended on intracellular TRF and HCO_3_^–^, and was further strengthened by exogenous addition of apo-TRF (Fig. 4, *A–D*, and Fig. S4, *A–D*). Indeed, TRF was clearly expressed in RPE and photoreceptor cells (Fig. 3*A*, and Fig. S3, *A* and *C*). There is already evidence that atRAL reacts with PE in photoreceptor outer segments to give A2PE, which is subsequently cleaved by phospholipase D (PLD) to produce A2E (33, 48). Therefore, it was easy to understand that A2E, different from atRAL-dimer, was not directly formed in reaction mixtures of atRAL, apo-TRF and NaHCO_3_ as well as atRAL, apo-TRF, DP-PE and NaHCO_3_ (Figure 1, Figure 2). DP-A2PE, a representative precursor of A2E, was generated in reaction mixtures of atRAL, DP-PE and NaHCO_3_, and its levels were significantly enhanced by apo-TRF (Fig. 2). Nevertheless, the production of A2E was clearly observed in primary pRPE cells or ARPE-19 cells after exposure to atRAL (Fig. 4, *C* and *D*, and Fig. S4, *C* and *D*), which is probably due to the inclusion of intracellular PE and PLD. Consistent with the findings that apo-TRF stimulated the formation of atRAL-dimer and DP-A2PE in the cell-free reactions (Figure 1, Figure 2), the amounts of atRAL-dimer and A2E in atRAL-loaded primary pRPE cells and ARPE-19 cells were also elevated by exogenous addition of apo-TRF (Fig. 4, *C* and *D*, and Fig. S4, *C* and *D*).

atRAL itself is not a transcriptionally active molecule, whereas all-*trans* retinoic acid (atRA) is a potent agonist of retinoic acid nuclear receptors at low nanomolar concentrations (49). We have previously reported that upon incubation of ARPE-19 cells with atRAL, a portion of atRAL is oxidized to atRA (50). To examine the effect of atRA and atRAL on the expression of *retinoid X receptor α* (*RXRα*) and *retinoic acid receptor β* (*RARβ*) genes regulated by atRA as well as *TRF* gene, primary pRPE cells and ARPE-19 cells were treated for 6 h with 40 and 15 μM of atRA or atRAL, respectively. The results showed that treatment with atRA significantly elevated mRNA levels of *RXRα* and *RARβ* in primary pRPE cells and ARPE-19 cells but it did not affect the expression of *TRF* gene (Fig. S9). By contrast, exposure to atRAL had no influence on the expression of *RXRα* and *RARβ* genes in primary pRPE cells and ARPE-19 cells yet it distinctly increased mRNA levels of *TRF* (Fig. S9), suggesting that the amount of atRA generated from the oxidation of atRAL in atRAL-loaded RPE cells is not enough for activating retinoic acid nuclear receptors. To monitor the impact of atRA on protein levels of TRF in RPE cells, primary pRPE cells and ARPE-19 cells were incubated for 6 h with 40 and 15 μM of atRAL or atRA, respectively. Immunoblot analysis of cell lysates demonstrated that atRA, different from atRAL, was incapable of facilitating the expression of TRF protein in RPE cells (Fig. S10).

ABCA4 and RDH8 function to clear free atRAL in the visual (retinoid) cycle, and their combined deficiencies are considered to be closely related to STGD1 and dAMD (1). Previous studies have documented that mice with defects in the function of ABCA4 and RDH8 exhibit a significant increase in levels of atRAL in the retina, which is further aggravated by exposure to light due to a strengthened release of atRAL from bleached rhodopsin (10, 13). The outcome of an excess of atRAL in the retina of mice is the death of photoreceptor and RPE cells (12). *Abca4*^*−/−*^*Rdh8*^*−/−*^ mice in response to light display an acceleration in retinal degeneration and elevation of atRAL levels in the retina compared to light-free *Abca4*^*−/−*^*Rdh8*^*−/−*^ mice (10, 12, 13), thereby enabling them to be an ideal acute model for investigating the pathogenesis of STGD1 and dAMD, and their therapy with drugs.

In serum of healthy people, the concentration of TRF ranges from two to 3 mg/ml (51), and the HCO_3_^–^ concentration is maintained at about 25 mM (52). It is also reported that in serum of C57BL/6J mice, the concentration of TRF is around 2 mg/ml (51), and the HCO_3_^–^ concentration is kept at about 22 mM (53). Therefore, the concentrations of apo-TRF and HCO_3_^–^ used in this study was of physiological significance. TRF is extremely abundant in neural retina and the RPE, and its mRNA levels in humans are more than six times greater in the retina than in the liver and cerebral cortex (30, 31, 54), which reflects that the unusually high expression of TRF in the retina likely has a unique retinal function (32). Chowers and colleagues disclose that TRF is upregulated in the retina from patients with dAMD(32). Using cell-based assays, we demonstrated that atRAL significantly elevated the expression of TRF in primary pRPE cells, ARPE-19 cells and 661W photoreceptor cells (Fig. 3*B*, and Fig. S3, *B* and *D*). Likewise, clearly increased expression of TRF was observed in neural retina and the RPE/choroid from light-exposed *Abca4*^*−/−*^*Rdh8*^*−/−*^ mice (Figure 5, *C* and *D*), probably because light exposure accelerates the release of atRAL from rhodopsin and thereby enhances its levels in the retina of *Abca4*^*−/−*^*Rdh8*^*−/−*^ mice. However, the present study showed that apo-TRF visibly ameliorated the viability and morphology of atRAL-loaded RPE cells by its ability to dimerize atRAL to generate atRAL-dimer and A2E (Fig. 4 and Fig. S4). More importantly, intravitreal injection of apo-TRF effectively rescued retinal function and alleviated retinal atrophy in *Abca4*^*−/−*^*Rdh8*^*−/−*^ mice after exposure to light (Fig. 7), and it clearly increased the production of atRAL-dimer and A2E in posterior eyecups (Fig. 6*D*). Moreover, exogenous TRF was clearly present in neural retina and RPE/choroid of *Abca4*^*−/−*^*Rdh8*^*−/−*^ mice intravitreally injected with apo-TRF, as well as apo-TRF-treated RPE cells (Figure 4, Figure 6, *A* and *B*, and Fig. S4, *A* and *B*). After injection of apo-TRF into the vitreous of mice, TRFr1 on the surface of photoreceptor and RPE cells and clathrin-mediated endocytosis may allow TRF into the cells (28, 29). In Table S1, we showed that albumin did not mediate the formation of atRAL-dimer with the help of HCO_3_^–^. As expected, retinal function decline and RPE degeneration were not prevented by intravitreal injection of 150 μg albumin (2 μl, 75 mg/ml in saline) into light-exposed *Abca4*^*−/−*^*Rdh8*^*−/−*^ mice (Fig. S11). Moreover, we tested two doses (50 and 150 μg; 2 μl, 25 and 75 mg/ml in saline) of apo-TRF for injections into the vitreous of light-exposed *Abca4*^*−/−*^*Rdh8*^*−/−*^ mice. The results of ERG and fundus imaging manifested that intravitreal administration of 50 and 150 μg apo-TRF showed a definite therapeutic effect on retinal function decline and RPE degeneration in *Abca4*^*−/−*^*Rdh8*^*−/−*^ mice upon exposure to light (Fig. S11). However, intravitreal injection of 150 μg apo-TRF exhibited a more pronounced therapeutic effect on the loss of retinal function and the dystrophy of RPE in light-exposed *Abca4*^*−/−*^*Rdh8*^*−/−*^ mice than that of 50 μg apo-TRF (Fig. S11). Taken together, these findings indicate that TRF exhibits a protective effect on retinal injury induced by atRAL through facilitating its dimerization, and suggest that the increase of TRF expression by atRAL serves as a mechanism of retinal self-protection.

A previous report has shown that each murine retina contains approximately 4.62 mM rhodopsin in disk membrane and 8.23 mM with respect to the rod outer segment cytoplasm (55). In addition, the bleaching of rhodopsin by light gives rise to an equivalent amount of atRAL (56). Hence, the atRAL concentration used in the current study could have physiological significance.

There are several reports suggesting that retinal dystrophies caused by high levels of atRAL or excess accumulation of its condensation products, especially A2E, are capable of being treated pharmacologically through inhibiting the visual (retinoid) cycle, limiting the supply of vitamin A or reacting with aldehyde groups (1). By contrast, the current approach was based on the new role of TRF as a mediator of atRAL self-condensation, and it directly targeted free atRAL and did not intervene in the operation of the visual (retinoid) cycle. TRF levels are higher in the periphery of the retina than in the macular area but in the human eye, the levels of A2E are highest in the far periphery and decrease toward the central region (32, 57), thus implying that the distribution of A2E positively correlates with TRF levels. Moreover, a previous study indicates that the correlation between A2E and lipofuscin is minimal in the human eye (58). A2E is derived from atRAL condensation reactions in the retina yet it is not mainly distributed in the lipofuscin of human eye, which supports the idea that endogenous production of A2E has a special function for the retina.

To sum up, our data lend support to the conclusion that TRF effectively prevented retinal function decline and degeneration caused by atRAL through mediating the dimerization of atRAL. This is the first report on the involvement of a protein in atRAL condensation reactions leading to the formation of bisretinoids in the retina. TRF could be developed as a promising drug candidate for the therapy of atRAL-associated retinopathies, such as STGD1 and dAMD.

## Experimental procedures

### Reagents and antibodies

atRAL (catalog no. R2500), atRA (catalog no. 1674004), NaHCO_3_ (catalog no. S6014), Na_2_HPO_4_ (catalog no. S9763), Na_2_SO_4_·10H_2_O (catalog no. 403008), apo-transferrin (apo-TRF) human (catalog no. T2252), holo-transferrin (holo-TRF) human (catalog no. T4132), albumin (catalog no. A6608), DP-PE (catalog no. P1348) and rabbit anti-TRF antibody (catalog no. HPA001527) were purchased from Sigma-Aldrich (Saint Louis, MO, USA). Mouse ZO-1 monoclonal antibody (catalog no. 33–9100) was provided by Invitrogen. β-actin (catalog no. 8457S) was obtained from Cell Signaling Technology. HRP-conjugated goat anti-rabbit IgG (H  + L) (catalog no. 31460), and Alexa Fluor 488-conjugated donkey anti-mouse IgG (H  + L) (catalog no. A21202) secondary antibodies were purchased from ThermoFisher Scientific (Rockford, IL, USA). H&E staining kit (catalog no. P032IH) was obtained from Auragene Biotech. ReverTra Ace qPCR RT Master Mix was purchased from TOYOBO Bio-Technology. PrimeSTAR HS (Premix) (catalog no. R040 A) was provided by Takara Biomedical Technology. FastStart Essential DNA Green Master was purchased from Roche Applied Science.

### Cell lines and culture conditions

Primary pRPE cells from freshly slaughtered pigs’ eyes were prepared as we previously described (27). The human RPE cell line ARPE-19 was purchased from FuDan IBS Cell Center (Shanghai, China). Murine photoreceptor cell line 661W was obtained from Shanghai Zishi Biotechnology (Shanghai, China). Primary pRPE cells, ARPE-19 cells or 661W photoreceptor cells were cultured in 1% penicillin/streptomycin containing DMEM supplemented with or without 10% fetal bovine serum (HyClone, Beijing, China) in a humidified incubator with 5% CO_2_ at 37 °C.

### Mice

*Abca4*^*−/−*^*Rdh8*^*−/−*^ mice on a C57BL/6J genetic background, which has a CTG (leucine) at codon 450 in the *Rpe65* gene and lacks *rd8* mutation in the *Crb1* gene, were generated and genotyped as we previously described (17, 18, 21). C57BL/6J mice were purchased from the Xiamen University Laboratory Animal Center. All experiments on mice were approved by the Institutional Animal Care and Use Committee of Xiamen University School of Medicine. *Abca4*^*−/−*^*Rdh8*^*−/−*^ and C57BL/6J mice at 4 weeks of age were dark adapted for 48 h. After pupils of dark-adapted mice were dilated with 1% tropicamide, the mice were illuminated for 1 h by white light-emitting diode (LED) light with an intensity of 10,000 lx and then placed in the dark for 5 days. Control *Abca4*^*−/−*^*Rdh8*^*−/−*^ and C57BL/6J mice were maintained normally in the dark for 7 days without light exposure. Retinal function of the mice was assessed by ERG. The mice were euthanized and their eyeballs were harvested for subsequent studies. Alternatively, the 48-h dark-adapted *Abca4*^*−/−*^*Rdh8*^*−/−*^ mice aged 4 weeks were anesthetized with 200 μl 1% pentobarbital sodium (100 μl/10 g), and then 2 μl of apo-TRF solution (25 and 75 mg/ml in saline; 50 and 150 μg apo-TRF), albumin solution (75 mg/ml in saline; 150 μg albumin) or saline (vehicle) were administered by intravitreal injection. Four hours later, pupils of the mice were dilated with 1% tropicamide, and the mice were exposed to 10,000 lx LED light for 1 h and then housed in the dark for additional 4 days. Control *Abca4*^*−/−*^*Rdh8*^*−/−*^ mice were injected intravitreally with apo-TRF or saline without light exposure. Retinal function and fundus morphology were detected by ERG and a small animal retinal imaging system (Optoprobe; OPIMG-L), respectively. The mice were euthanized and their eyeballs were collected for the following experiments.

### Synthesis of A2E, isoA2E and atRAL-dimer

A2E, isoA2E and atRAL-dimer were synthesized as previously described (23, 24).

### Reactions of atRAL with apo-TRF

atRAL was first dissolved in dimethyl sulfoxide (DMSO) to prepare a 10 mM stock solution. Then 2 μl of the atRAL stock solution (10 mM in DMSO) was added into 500 μl water containing apo-TRF (0.5, 1, two or 4 mg/ml) and 24 mM NaHCO_3_. When mixed evenly, the solution was clear with an atRAL concentration of 40 μM. Next, it was incubated for 6 h, 12 h, 1 day, 3 days and 5 days at 37 °C in an incubator with 5% CO_2_ in the dark. The reaction mixtures were dried under argon gas and then re-dissolved in 100 μl methanol. After being centrifuged at 13,400 *g* for 10 min, the supernatant was subjected to reverse-phase HPLC using a Waters Alliance System (Milford, MA, USA) equipped with a 2695 separation module, a 2998 photodiode array detector, and a 2475 multichannel (λ) fluorescence detector. An Atlantis dC18 reverse-phase column (3 μm, 4.6 × 150 mm), operating at 35°C, was used for the stationary phase. Compounds were eluted with a gradient mobile phase consisting of acetonitrile and water with 0.1% trifluoroacetic acid (TFA): 85−100% acetonitrile, 0.8 ml/min, 15 min; 100% acetonitrile, 0.8−1.2 ml/min, 15−20 min; 100% acetonitrile, 1.2 ml/min, 20−40 min. The peak area (microvolts per second, μV/s) was integrated using Empower version 3 software. Photodiode array detection was set at 430 nm.

### Reactions of atRAL with DP-PE in the presence of apo-TRF

Two microliters of the atRAL stock solution (10 mM in DMSO) were added into 500 μl water containing 4 mg/ml apo-TRF, 800 μM DP-PE and 24 mM NaHCO_3_. When mixed evenly, the solution was clear with an atRAL concentration of 40 μM. Subsequently, it was incubated for 5 days in an incubator at 37 °C with 5% CO_2_ in the dark. After the solvent was completely evaporated under argon gas, the residues were dissolved using 100 μl of methanol and then centrifuged at 13,400 *g* for 10 min. For HPLC analysis, the supernatant was injected into an Atlantis dC4 reverse-phase column (5 μm, 3.9 × 150 mm). The mobile phase was the same as described above for HPLC. The photodiode array detector was set at 430 nm for eluent monitoring.

### UPLC/APCI-MS

UPLC–MS was performed using an AB Sciex Qtrap 6500+ mass spectrometer coupled on-line to a Waters Acquity UPLC I-Class. The mass spectrometer was equipped with an APCI interface and ion trap analyzer operating in full scan mode from *m/z* 50 to 2000. The same dC18 column and mobile phase were used as described above for HPLC. Mass spectra were achieved using APCI in positive ion mode or in MS/MS mode. Analyst software was used for data acquisition and instrument control.

### HPLC/ESI-HRMS

HPLC-HRMS was carried out using a benchtop Q-Exactive Orbitrap high-resolution mass spectrometer (Thermo Scientific; Waltham, MA, USA) that was coupled on-line to a Thermo Scientific Ultimate3000 HPLC. The mass spectrometer was equipped with ESI interface and ion trap analyzer operating in full scan mode from *m/z* 50 to 2000. The same dC4 column and mobile phase were used as described above for HPLC. Mass spectral detection was achieved using ESI in positive ion mode. Thermo Scientific Xcalibur software permitted data acquisition and instrument control.

### Treatment with atRAL or atRA

Primary pRPE cells, ARPE-19 cells or 661W photoreceptor cells were seeded into 6-well plates and cultured overnight. Primary pRPE cells were exposed for 6 h to 40 μM atRAL or atRA. ARPE-19 cells were incubated for 6 and 12 h with 15 μM atRAL, or treated for 6 h with 15 μM atRA. 661W photoreceptor cells were treated for 6 h with 5 μM atRAL.

### Cell viability and morphology

Primary pRPE cells or ARPE-19 cells were seeded into 96-well plates and cultured overnight. Primary pRPE cells were pretreated with apo-TRF (2 mg/ml in DMEM) for 3 days and then incubated with 40 μM atRAL for 1 day. ARPE-19 cells were preincubated with apo-TRF (2 mg/ml in DMEM) for 3 days and then exposed for 1 day to 15 μM atRAL. It should be mentioned here that apo-TRF-treated RPE cells were washed 3 times with PBS prior to incubating with atRAL. Cytotoxicity was detected by MTS assay as we previously reported (17). Cellular morphology was photographed under a Leica DM2500 microscope (Wetzlar, Germany).

### H&E staining

The eyeballs of mice were fixed in formaldehyde, acetic acid and saline fixative solution (Servicebio) at 4 ^o^C for 24 h and then embedded in paraffin. Sections at the thickness of four microns were cut from paraffin-embedded tissues and routinely stained with H&E. Photographs were taken with the Leica DM2500 microscope (Wetzlar, Germany).

### RPE tight junctions

Eyeballs of mice were fixed for 30 min in 4% paraformaldehyde at room temperature. Posterior eyecups containing RPE/choroids were carefully obtained and then penetrated with 1% Triton X-100 for 1 h, followed by incubation in PBS containing 5% bovine serum albumin and 0.3% Triton X-100 for 1 h. RPE/choroid flat mounts were stained with a mouse monoclonal antibody against ZO-1 at a dilution of 1:100 overnight at 4°C, and after being washed by PBS three times, they were incubated with an Alexa Fluor 488-conjugated donkey anti-mouse IgG (H  + L) secondary antibody (1:200 dilution) for 2 h. Images were captured by an Olympus FV1000 confocal microscope (Tochigi, Japan).

### Treatment with apo-TRF

Primary pRPE cells or ARPE-19 cells were sowed into 100 mm plates and cultured overnight, followed by complete replacement of the culture medium by fresh DMEM without serum. Cells were treated with apo-TRF (2 mg/ml in DMEM) for 12 h, 1 day or 3 days, and completely washed with PBS to ensure that only intracellular TRF remained. Alternatively, primary pRPE cells and ARPE-19 cells were exposed for 3 days to 2 mg/ml apo-TRF, and then incubated for 1 day with 40 and 15 μM atRAL, respectively. After completely washing the cells with PBS, the lysates from the same number of cells were extracted with 50% methanolic chloroform. By centrifugation at 3900 *g* for 5 min, the organic layer was collected in a 25 ml round-bottom flask. Extraction was repeated with chloroform three times, and the extracting solution was combined into the 25 ml round-bottom flask. Organic solvents were removed by a rotary evaporator. By using 50% methanolic chloroform, residues were transferred into a 0.5 ml eppendorf tube and dried under argon gas. The resulting extract was dissolved in 100 μl of methanol, and injected into the reverse-phase HPLC. For the elution of compounds from the dC18 column, the following gradient of acetonitrile in water with 0.1% TFA was utilized: 0−30 min, 75% acetonitrile, 0.5 ml/min; 30−40 min, 90% acetonitrile, 0.5 ml/min; 40−100 min, 100% acetonitrile, 0.5 ml/min. Photodiode array detection was set at 430 nm.

### Quantitative real-time PCR

Total cellular RNA was extracted using TRIeasy total RNA extraction reagent. Concentration, purity, and integrity of total RNA were determined by NanoDrop One (Thermo Fisher Scientific). One-μg RNA was reverse transcribed into complementary DNA using ReverTra Ace qPCR RT Master Mix following the manufacturer’s protocol. qRT-PCR was performed on a LightCycler 96 instrument (Roche Applied Science) using the FastStart Essential DNA Green Master. Primer sequences were available in Table S2.

### Immunostaining

Primary pRPE cells or ARPE-19 cells seeded on cover slips in 6-well plates were incubated with 2 mg/ml apo-TRF or DMEM alone for 3 days. After being fixed in 4% paraformaldehyde at 4 ^o^C for 15 min, cells were permeabilized by 0.2% Triton X-100 in PBS for 20 min and then immersed in 2% bovine serum albumin for 1 h to abolish the nonspecific binding at room temperature. Next, cells were incubated with anti-TRF antibody (1:100 dilution) and mouse monoclonal antibody against ZO-1 (1:100 dilution) at 4 ^o^C overnight, followed by incubation with Alexa Fluor 594-conjugated donkey anti-rabbit secondary antibody (1:100 dilution) and Alexa Fluor 488-conjugated donkey anti-mouse IgG (H  + L) secondary antibody (1:200 dilution) for 2 h at room temperature. Slides were mounted with DAPI in 50% glycerin. Photographs were taken using a Zeiss LSM 880+airy scan confocal microscope (Carl Zeiss; Jena, Germany).

### Western blotting

Neural retina and RPE/choroid were dissected from mouse eyeballs, respectively. Western blot analysis of extracts from cells or tissues was performed as we previously described (17). Equal quantities of protein were subjected to electrophoresis in 12% SDS-polyacrylamide gels and transferred to polyvinylidene difluoride membranes (Roche Applied Science). The membranes, 1 h after being blocked with 5% skim milk at room temperature, were incubated with primary antibodies (1: 1000 dilution) specific for TRF or β-actin at 4 ^o^C overnight, followed by incubation with the corresponding secondary antibodies (1: 5000 dilution) for 1 h at room temperature. Blots were imaged on a ChemiDoc XRS + Imaging system (Bio-Rad) by using electrochemiluminescence Western blotting detection reagents (Advansta). Band intensities were quantified by densitometry using ImageJ software. Unprocessed original images of gels, in which boxes in red indicated selected Western blot results, were shown in Figure S12.

### Fundus imaging

Mice were anesthetized with 1% pentobarbital sodium (100 μl/10 g) by intraperitoneal injection, and their pupils were dilated with 1% tropicamide. Next, 0.2% carbomer solution was used for preventing cataract formation and keeping the cornea surface clarified and hydrated. Fundus images of the mice were acquired by a small animal retinal imaging system (Optoprobe; OPIMGL, UK).

### Intravitreal injection

Mice were intraperitoneally anesthetized with 1% pentobarbital sodium (100 μl/10 g), and their pupils were dilated with 1% tropicamide. Following the dropping of 0.2% carbomer solution into eyes, a small scleral preincision was made behind the corneoscleral rim using a 30G needle under a stereomicroscope. A 33 G needle filled with 2 μl apo-TRF solution (25 and 75 mg/ml in saline; 50 and 150 μg apo-TRF), albumin solution (75 mg/ml in saline; 150 μg albumin) or saline (vehicle) was then precut from the corneal limbus relative to the optic nerve, and inserted about 1 mm deep into the vitreous cavity, followed by a smooth release of apo-TRF, albumin or saline through operating the syringe. To avoid reflux out of the injection site, the needle was retrieved very slowly.

### ERG

Mice, which were dark adapted for 8 h, anesthetized with a low flow rate of isoflurane continuously through their nose and mouth, and then subjected to a computer-based system (RetiMINER IV, China IRC Medical Equipment Co., Ltd) for ERG recordings in response to flashes produced with LEDs or Xenon bulbs. Corneal ERGs were recorded from both eyes of each mouse using two gold wire loop electrodes. Moreover, a needle electrode was inserted subcutaneously into the posterior neck, and a ground electrode was connected to the tail. For scotopic ERG, mice were stimulated with flashes of increasing light intensity (from 0.001 to 10 cd s/m^2^). For photopic ERG, mice were stimulated with flashes of increasing light intensity (from 1 to 10 cd s/m^2^). Responses were computer averaged and recorded at 3 to 60 s intervals depending on the stimulus intensity.

### Tissue extraction and HPLC analysis

Murine posterior eyecups (2–8 eyes/sample) were homogenized in 0.5 ml water by using a SCIENTZ-48 high-throughput tissue grinder (SCIENTZ; Ningbo, China). The mixture was transferred to a 15 ml conical centrifuge tube and then extracted with 50% methanolic chloroform. Following centrifugation at 3000*g* for 5 min, the organic layer was placed in a 25 ml round-bottom flask. Extraction was repeated with chloroform three times, and the extracting solution was combined into the round-bottom flask. After the removal of combined solvents in a rotary evaporator, the residues were transferred into a 0.5 ml centrifuge tube with 50% methanolic chloroform and dried under argon gas. The resulting extract was dissolved in 100 μl methanol and centrifuged at 13,400 *g* for 10 min, followed by examination of the supernatant by the reverse-phase HPLC. The analytical scale Atlantis dC18 column (3 μm, 4.6 × 150 mm) was used for chromatographic separation. Compounds were eluted with a gradient mobile phase consisting of acetonitrile and water in the presence of 0.1% TFA: 75 to 90% acetonitrile (0–30 min), 90 to 100% acetonitrile (30–40 min), and 100% acetonitrile (40–100 min) with a flow rate of 0.5 ml/min. Photodiode array detection was set at 430 nm.

### Statistical analysis

All data from at least three independent experiments were analyzed using GraphPad Prism software (Version 8.0; La Jolla, CA), and presented as mean ± standard deviation (SD). Statistical analyses were performed using Student’s *t* test, or one-way or two-way analysis of variance (ANOVA) followed by Tukey’s multiple comparison test or Bonferroni's multiple comparison test, as shown in corresponding figure legends. In all cases, *p*-values below 0.05 were considered statistically significant.

## Data availability

The data supporting the findings of this study are available within the article and the supporting information.

## Supporting information

This article contains supporting information.

## Conflicts of interest

The authors declare that they have no conflicts of interest with the contents of this article.
