# Supplementary material for: Transferrin ameliorates retinal degeneration by mediating the dimerization of all-trans-retinal
Source: J Biol Chem. 2024 Dec 9;301(1):108054. doi: 10.1016/j.jbc.2024.108054 (PMC11742617; doi:10.1016/j.jbc.2024.108054)
Supplement: Supplementary Figures and Tables [file mmc1.pdf]

Supplementary Information

Transferrin ameliorates retinal degeneration by mediating the dimerization of all-*trans*-retinal

Lei Tao[1,2], Danxue He[1,2], Yuling Chen[1,2], Kunhuan Yang[1,2], Beiting He[1,2], Peixin Cai[1,2], Binxiang Cai[1,2], Chunyan Liao[1,2], Zuguo Liu[2], Shiying Li[1], Jingmeng Chen[3,4], and Yalin Wu[1,2,4,*]

[1]Department of Ophthalmology, the First Affiliated Hospital of Xiamen University, School of Medicine, Xiamen University, Xiamen, Fujian 361003, China
[2]Fujian Provincial Key Laboratory of Ophthalmology and Visual Science, Fujian Engineering and Research Center of Eye Regenerative Medicine, Eye Institute of Xiamen University, School of Medicine, Xiamen University, Xiamen, Fujian 361102, China
[3]School of Medicine, Xiamen University, Xiamen, Fujian 361102, China
[4]Shenzhen Research Institute of Xiamen University, Shenzhen, Guangdong 518063, China
*Correspondence: yalinw@xmu.edu.cn (Y. W.)

Table S1

Screening of endogenous proteins involving the conversion of atRAL into atRAL-dimer

All proteins were purchased from Sigma-Aldrich (Saint Louis, MO, USA). Each protein at the concentration of 0.5 mg/ml was incubated with 40 µM atRAL and 24 mM $NaHCO_3$ in 500 µl water for 1 day at 37°C in an incubator with 5% CO_2 in the dark. The reaction mixtures were dried under argon gas and then re-dissolved in 100 µl methanol. After being centrifuged at 13,400 *g* for 10 min, the supernatant was subjected to reverse-phase HPLC using a Waters Alliance System (Milford, MA, USA) equipped with a 2695 separation module, a 2998 photodiode array detector, and a 2475 multichannel (λ) fluorescence detector. An Atlantis® dC18 reverse-phase column (3 µm, 4.6×150 mm), operating at 35°C, was used for the stationary phase. Compounds were eluted with a gradient mobile phase consisting of acetonitrile and water with 0.1% TFA: 85−100% acetonitrile, 0.8 ml/min, 15 min; 100% acetonitrile, 0.8−1.2 ml/min, 15−20 min; 100% acetonitrile, 1.2 ml/min, 20−40 min.

Protein (0.5 mg/ml)	HCO_3^- (24 mM)	atRAL (40 µM)	atRAL dimerization
Apo-transferrin (Apo-TRF) human (catalog no. T2252)	+	+	+
Albumin (catalog no. A6608)	+	+	–
Fibrinogen (catalog no. F4883)	+	+	–
Prothrombin (catalog no. 539515)	+	+	–
Ferritin (catalog no. F6754)	+	+	–
Hemopexin (catalog no. H9291)	+	+	–
Insulin (catalog no. I3536)	+	+	–
Complement factor H (catalog no. C5813)	+	+	–
Retinol-binding protein 4 (RBP4) (catalog no. R9388)	+	+	–
NGAL (lipocalin-2) (catalog no. SRP4928)	+	+	–
Beta globulin (catalog no. 475828)	+	+	–
Gamma globulin (catalog no. G4386)	+	+	–
High density lipoprotein (HDL) (catalog no. L8039)	+	+	–
Glucagon (catalog no. 05-23-2700)	+	+	–
Lactic dehydrogenase （LDH) (catalog no. L7525)	+	+	–
C-reactive protein (catalog no. C4063)	+	+	–
Transthyretin (TTR) (catalog no. P1742)	+	+	–
Lactoferrin (catalog no. L4040)	+	+	–
Tumor necrosis factor-α (TNF-α) (catalog no. H8916)	+	+	–
Interleukin-1β (IL-1β) (catalog no. IL038)	+	+	–
Interleukin-6 (IL-6) (catalog no. SRP3096)	+	+	–
Transforming growth factor-β (TGF-β) (catalog no. GF313)	+	+	–
Growth hormone (catalog no. SRP6167)	+	+	–

Table S2
Primer sequences

Gene	Forward primer	Reverse primer
TRF **(mouse)**	GCTGTCCCTGACAAAACGGT	GTCACGGAAGCTGATGCACT
β-actin **(mouse)**	GATCAAGATCATTGCTCCTCCTG	AGGGTGTAAAACGCAGCTCA
TRF **(human)**	TGTGAGATGGTGTGCAGTGT	GCTTCGTTTGCCGCAATG
TRF **(pig)**	TCCGCAGAAAACACCGAAGA	AGCCACAGCCAAATACCCTTT
RXRα **(human)**	CTCCCTTCACTTCCTGGCCATC	GCCTCAGGTTTTCTCATCCGC
RARβ **(human)**	CCAAACCGAATGGCAGCATC	TCTGCGGAAAAAGCCCTTACA
RXRα **(pig)**	TCTTAACACCTGCACACTGGT	GTAGGGACAAGCACCCACTT
RARβ **(pig)**	GTCAGACTCGCTGGGTCATT	GCGGAAAAACCCCTTACAGC
β-actin **(human)**	TTCGCGGGCGACGAT	CACATAGGAATCCTTCTGACCC
Gapdh **(pig)**	GTCGGAGTGAACGGATTTGG	CACCCCATTTGATGTTGGCG

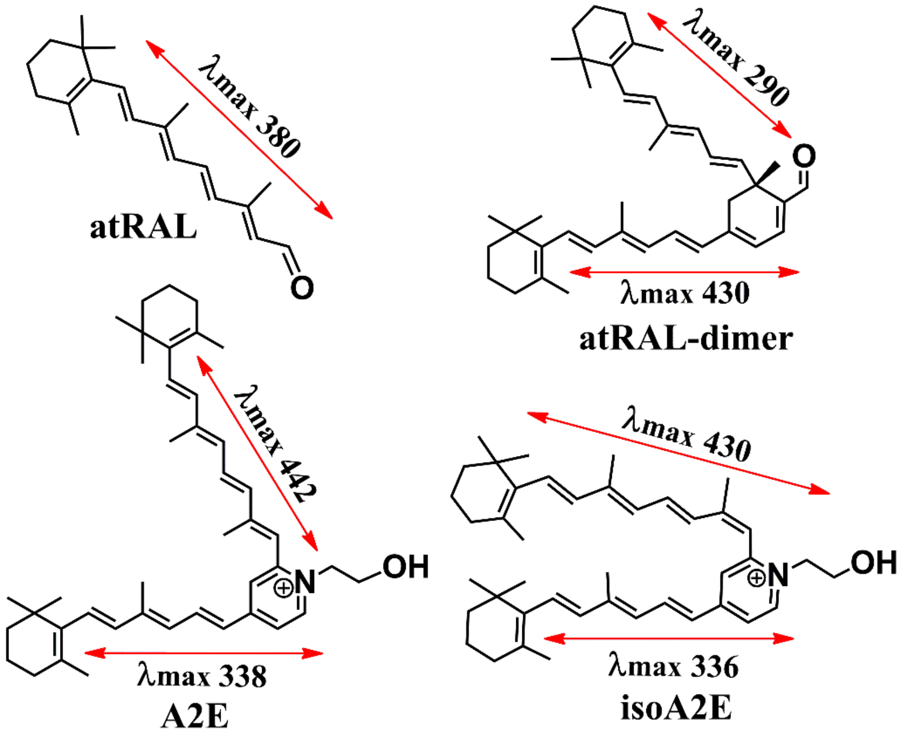

Figure S1. atRAL, atRAL-dimer, A2E and isoA2E. Shown are structures, UV-visible absorbance maxima (nanometers), and electronic transition assignments (↔).

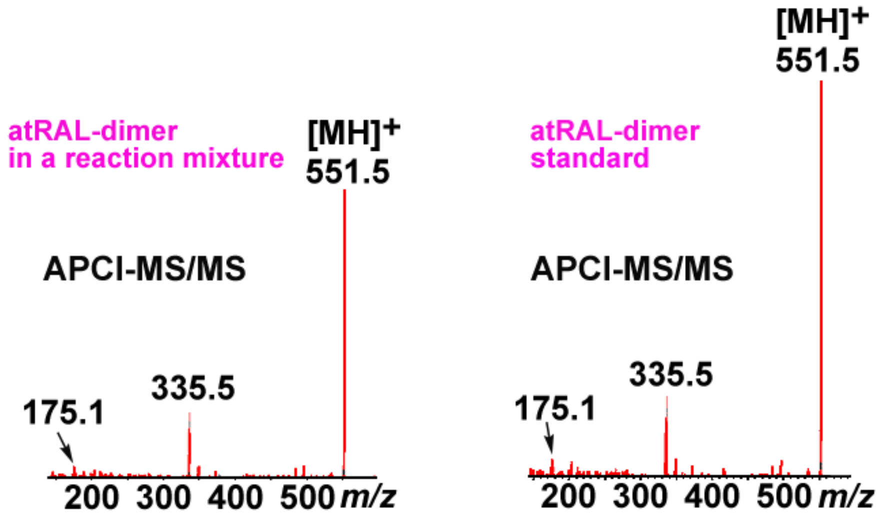

Figure S2. APCI-MS/MS analysis of atRAL-dimer generated from reaction of atRAL with apo-TRF and $NaHCO_3$, and atRAL-dimer standard. atRAL (40 µM) was reacted with apo-TRF (4 mg/ml) and $NaHCO_3$ (24 mM) at 37ºC in the dark for 1 day in 500 µl water. In addition to atRAL-dimer standard, atRAL-dimer in the reaction mixture was ionized by APCI in positive ion mode, and detected on an AB Sciex Qtrap 6500+ MS instrument in MS/MS mode using an Analyst software.

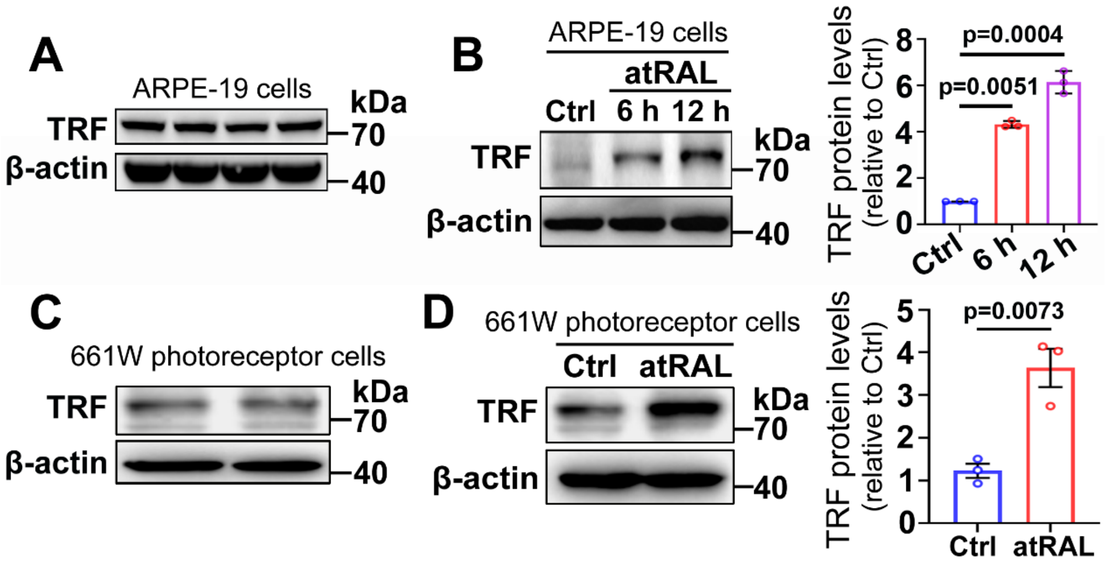

Figure S3. The expression of TRF is evident and it is significantly increased by atRAL in ARPE-19 cells or 661W photoreceptor cells. *A,* immunoblots of TRF in ARPE-19 cells. *B,* Western blots of TRF in ARPE-19 cells exposed for 6 and 12 h to 15 μM atRAL. Protein levels of TRF were normalized to those of β-actin and expressed as fold changes compared with DMSO-treated controls. *C,* immunoblots of TRF in 661W photoreceptor cells. *D,* Western blots of TRF in 661W photoreceptor cells incubated for 6 h with 5 μM atRAL. One-way ANOVA with Tukey's post-test in *B* and Student's *t*-test in *D* were carried out for statistical analyses.

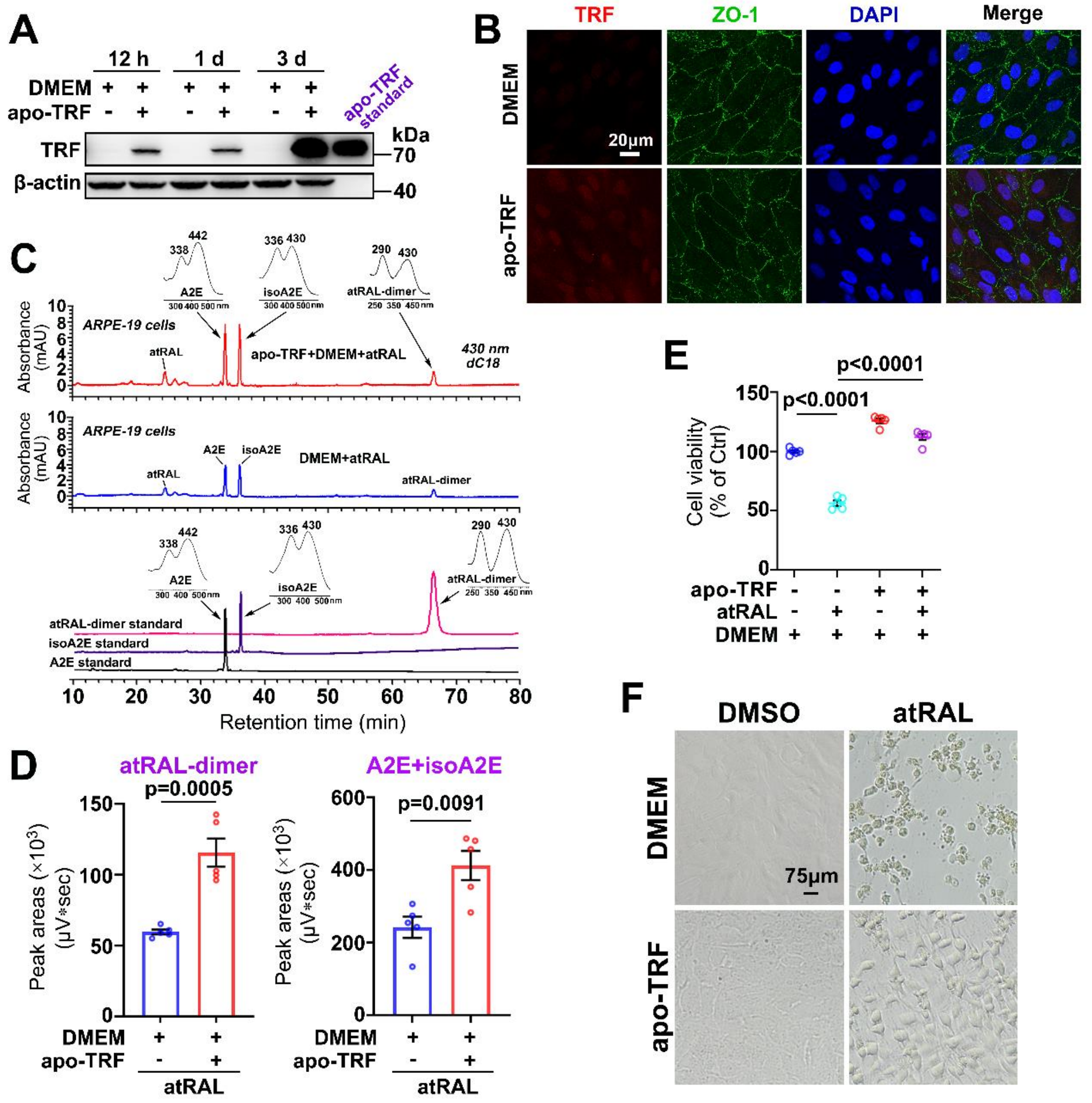

Figure S4. Treatment with apo-TRF reduces the cytotoxicity of atRAL in ARPE-19 cells through promoting the dimerization of atRAL. *A,* Western blots of TRF in ARPE-19 cells treated with or without 2 mg/ml apo-TRF for 12 h, 1 day and 3 days in DMEM. Commercial apo-TRF standard served as a positive control. *B,* internalization of apo-TRF was evaluated by immunofluorescence co-staining for TRF (*red*) and ZO-1 (*green*) using confocal laser scanning fluorescence microscopy. ARPE-19 cells were treated for 3 days with 2 mg/ml apo-TRF or DMEM alone. Nuclei were stained with DAPI (*blue*). *Scale bars,* 20 µm. *C,* typical HPLC chromatograms (dC18 column; 430 nm monitoring) were generated with the extracts from the same number of ARPE-19 cells that were pretreated with 2 mg/ml apo-TRF for 3 days and then exposed for 1 day to 15 µM atRAL in DMEM. Control cells were cultured in DMEM for 3 days, followed by 1 day of treatment with atRAL. Also shown were HPLC profiles of synthetic standards for A2E, isoA2E and atRAL-dimer. *Insets*, UV-visible absorbance spectra of A2E, isoA2E and atRAL-dimer. *D,* the levels of atRAL-dimer, A2E and isoA2E were quantified using Empower version 3 software. The amounts of A2E were calculated as the sum of the levels of A2E and isoA2E. Each value represents mean ± SD (n=5).

mAU, milliabsorbance unit. *E* and *F,* cell viability and cellular morphology were assessed by MTS assay and light microscopy, respectively. ARPE-19 cells were pretreated for 3 days with 2 mg/ml apo-TRF in DMEM, followed by 1-day incubation with or without 15 μM atRAL. Cells treated with atRAL or DMSO alone in DMEM serve as controls. Student's *t*-test in *D* and one-way ANOVA with Tukey's post-test in *E* were carried out for statistical analyses. d, day (s).

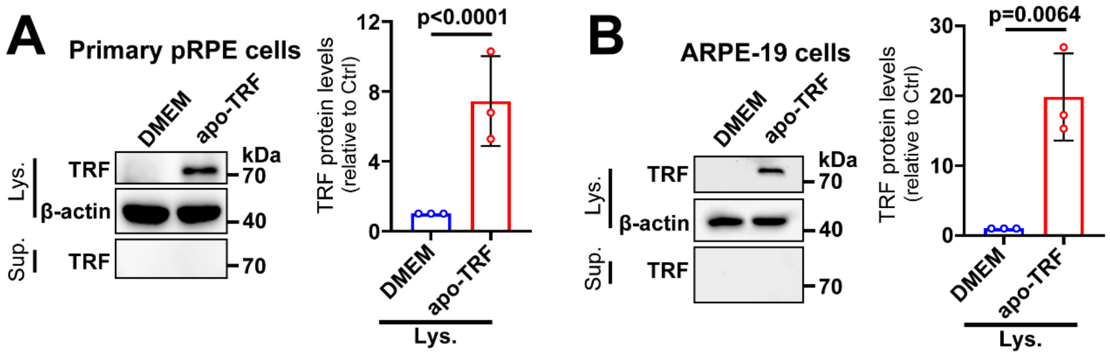

Figure S5. RPE cells carrying exogenous TRF are completely washed with phosphate buffer saline (PBS). Primary pRPE cells (*A*) or ARPE-19 cells (*B*) were incubated for 3 days with 2 mg/ml apo-TRF and DMEM alone, respectively. Cells were washed three times by PBS. Following the addition of fresh DMEM, the supernatant of cells was collected by centrifugation and examined by Western blotting. Moreover, cell lysates were also subjected to immunoblotting. *A,* Western blots of TRF in cell lysates and supernatant of primary pRPE cells incubated with apo-TRF or DMEM alone. *B,* immunoblots of TRF in cell lysates and supernatant of ARPE-19 cells exposed to apo-TRF or DMEM alone. Protein levels of TRF in cell lysates were normalized to those of β-actin, and shown as fold changes relative to DMEM-treated controls. Statistical analyses were assessed by Student's *t*-test. Lys., Lysate. Sup., Supernatant.

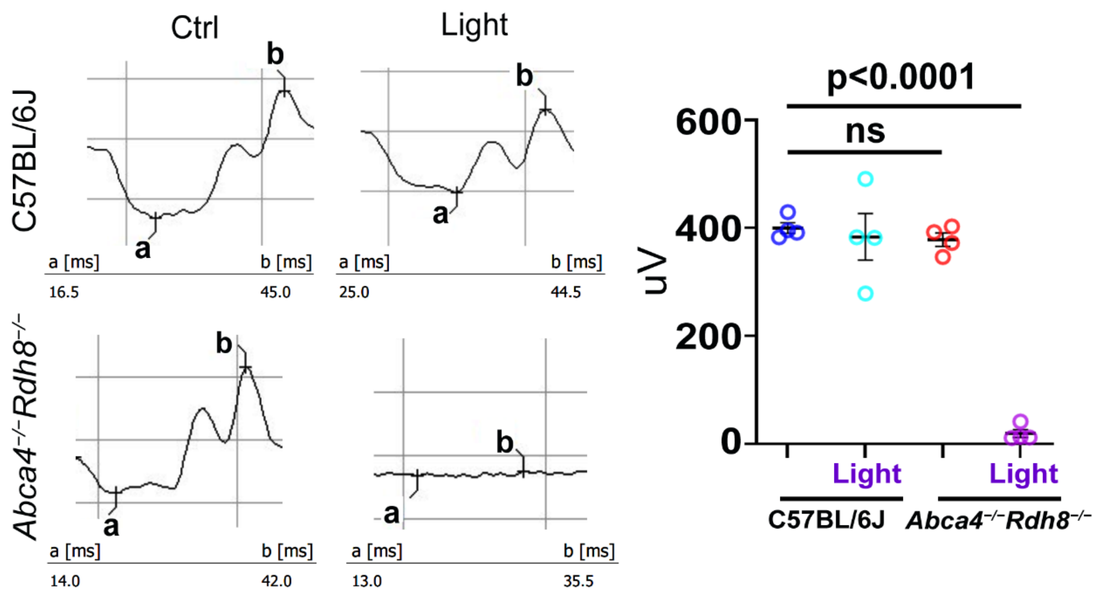

Figure S6. The effect of light exposure on retinal function in C57BL/6J and *Abca4*$^{-/-}$*Rdh8*$^{-/-}$ mice. C57BL/6J and *Abca4*$^{-/-}$*Rdh8*$^{-/-}$ mice at 4 weeks of age were placed in a dark room for 2 days. After pupils of dark-adapted mice were dilated with 1% tropicamide, the mice were exposed to 10,000 lx LED light for 1 h and then kept in the dark for 5 days. Control C57BL/6J and *Abca4*$^{-/-}$*Rdh8*$^{-/-}$ mice were maintained normally in the dark for 7 days in the absence of light exposure. Retinal function of the mice was examined by ERG. ERG amplitudes were quantified using a computer-based system. The differences in amplitudes of a- and b-waves were used to evaluate retinal function, and expressed as mean ± SD (n=4). µV, microvolt. Statistical analyses were conducted by one-way ANOVA with Tukey's post-test.

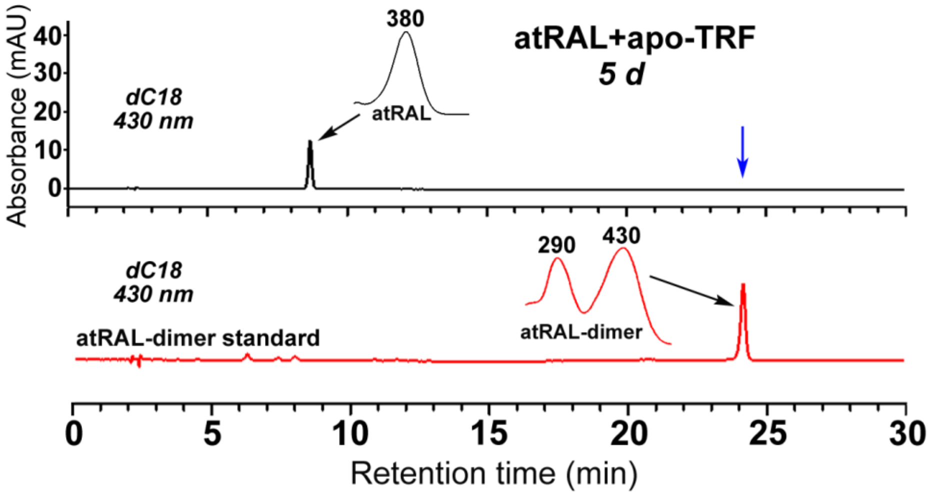

Figure S7. Incubation of atRAL with apo-TRF alone for 5 days does not produce atRAL-dimer. A typical HPLC chromatogram (dC18 column; 430 nm monitoring) was generated from reaction mixtures of atRAL (40 µM) and apo-TRF (4 mg/ml); incubation at 37°C in the dark for 5 days in 500 µl water. Also shown was a representative HPLC profile of atRAL-dimer standard. *Inset*s, UV-visible absorbance spectra of atRAL and atRAL-dimer. d, days.

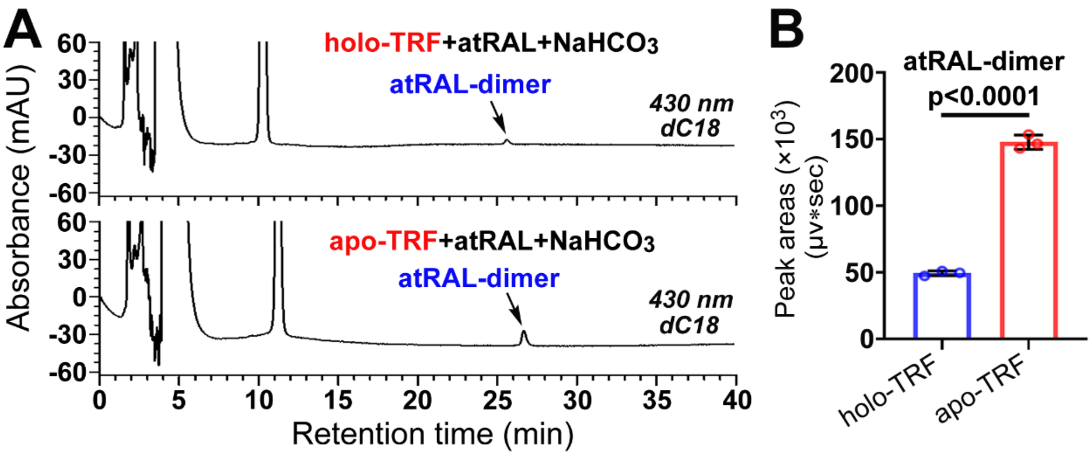

Figure S8. Comparison of the effect of apo-TRF and holo-TRF on the conversion of atRAL into atRAL-dimer with the help of HCO_3^-. *A,* representative HPLC chromatograms (dC18 column; 430 nm monitoring) of the mixtures from reactions of 4 mg/ml apo-TRF or holo-TRF with 40 µM atRAL and 24 mM $NaHCO_3$; incubations at 37ºC in the dark for 1 day in 500 µl water. The reaction mixtures were dried under argon gas and then re-dissolved in 100 µl methanol. After being centrifuged at 13,400 *g* for 10 min, the supernatant was subjected to the reverse-phase HPLC as described above. For the elution of compounds from the dC18 column, the following gradient of acetonitrile in water with 0.1% TFA was utilized: 85−100% acetonitrile, 0.8 ml/min, 15 min; 100% acetonitrile, 0.8−1.2 ml/min, 15−20 min; 100% acetonitrile, 1.2 ml/min, 20−40 min. *B,* the levels of atRAL-dimer were quantified using Empower version 3 software. Each value represents mean ± SD (n=3). mAU, milliabsorbance unit. Student's *t*-test was performed for statistical analyses.

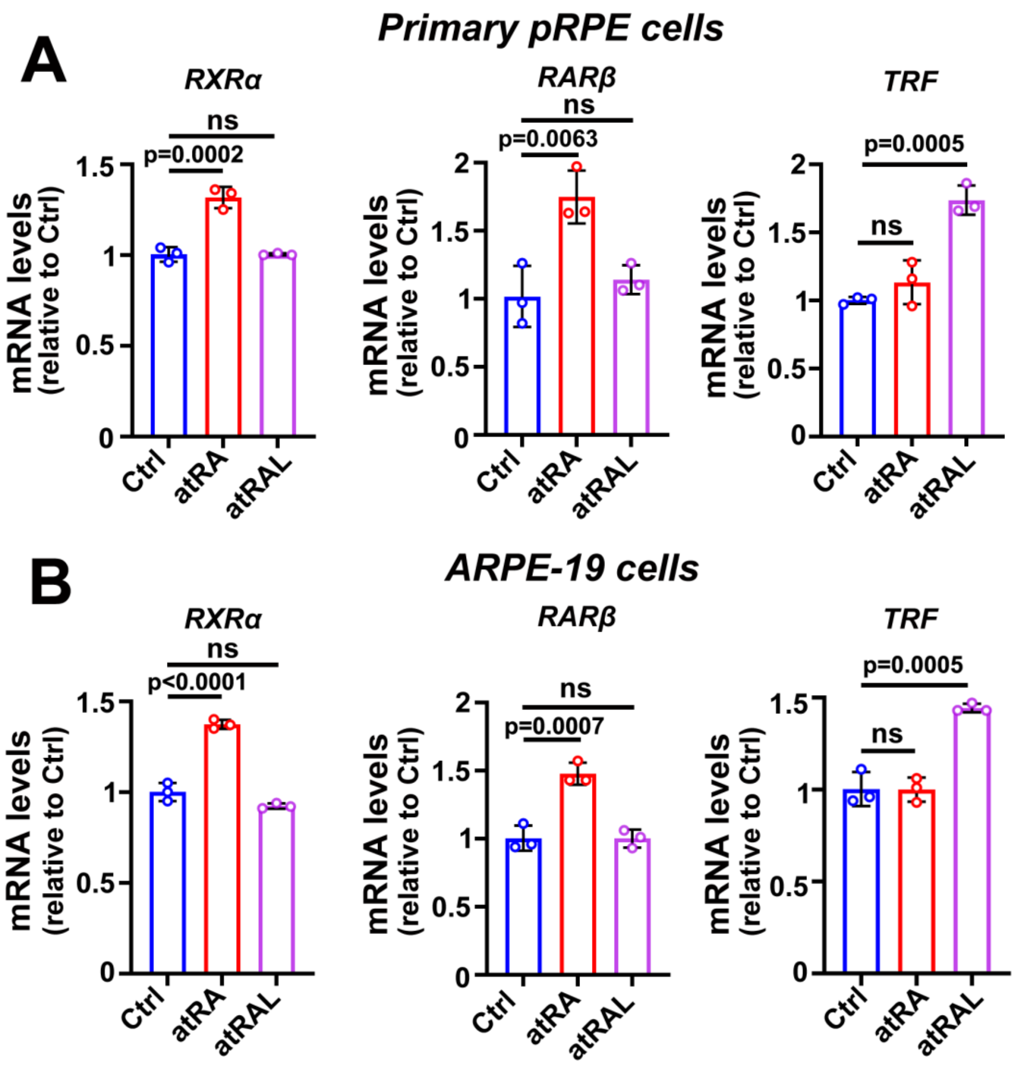

Figure S9. Effects of atRA and atRAL on the expression of *RXRα*, *RARβ* and *TRF* genes in primary pRPE cells or ARPE-19 cells. *A,* qRT-PCR analysis of *RXRα*, *RARβ* and *TRF* genes in primary pRPE cells incubated for 6 h with 40 μM atRA or atRAL. *B,* qRT-PCR was used to evaluate the expression of *RXRα*, *RARβ* and *TRF* genes in ARPE-19 cells exposed for 6 h with 15 μM atRA or atRAL. Control cells were treated with DMSO alone. The mRNA levels of these genes were shown as fold changes relative to DMSO-treated controls. Statistical analyses were conducted by one-way ANOVA with Tukey's post-test. ns, not significant.

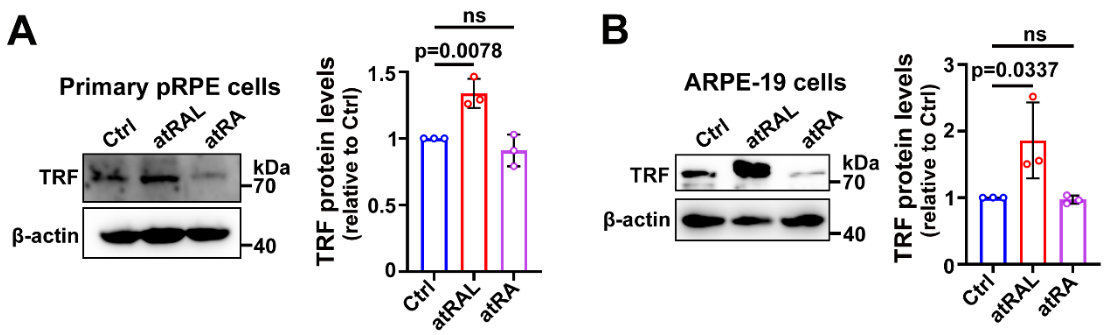

Figure S10. Treatment with atRA is unable to promote the expression of TRF protein in RPE cells. *A,* Western blots of TRF in primary pRPE cells incubated for 6 h with 40 μM atRAL or atRA. *B,* immunoblots of TRF in ARPE-19 cells treated for 6 h with 15 μM atRAL or atRA. Control cells were exposed to DMSO alone. Protein levels of TRF were shown as fold changes relative to DMSO-treated controls. Statistical analyses were carried out by one-way ANOVA with Tukey's post-test.

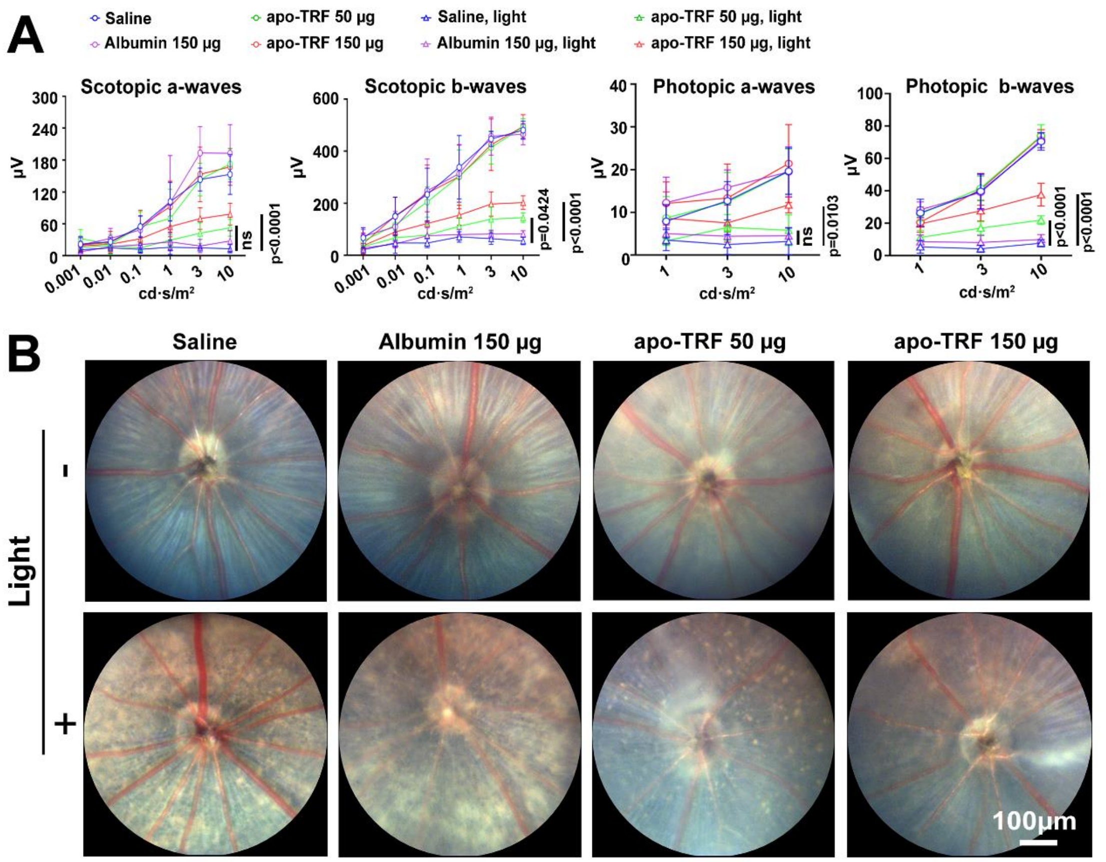

Figure S11. Therapeutic effects of apo-TRF and albumin on retinal function decline and RPE degeneration in light-exposed *Abca4*$^{-/-}$*Rdh8*$^{-/-}$ mice. *Abca4*$^{-/-}$*Rdh8*$^{-/-}$ mice at the age of 4 weeks were dark adapted for 48 h, and then intravitreally injected with 50 and 150 µg apo-TRF (2 µl, 25 and 75 mg/ml in saline), 150 µg albumin (2 µl, 75 mg/ml in saline), or vehicle (saline). Four hours later, the mice were illuminated with 10,000 lx LED light for 1 h after their pupils were dilated with 1% tropicamide. Control *Abca4*$^{-/-}$*Rdh8*$^{-/-}$ mice were administered intravitreally with albumin, apo-TRF or saline without exposure to light. *A,* retinal function of the mice was examined by ERG. ERG amplitudes of a- and b-waves, which were quantified using a computer-based system, were respectively used to evaluate retinal function and shown as mean ± SD (n=5). Statistical analyses were performed using two-way ANOVA with Tukey's post-test. µV, microvolt. ns, not significant. For scotopic ERG, mice were stimulated with flashes of increasing light intensity (from 0.001 to 10 cd·s/m²). Regarding photopic ERG, mice were stimulated with flashes of increasing light intensity (from 1 to 10 cd·s/m²). *B,* the RPE morphology of each mouse was visualized by a small animal retinal imaging system (Optoprobe; OPIMG-L, UK). *Scale bars,* 100 µm.

Figure S12. Unprocessed original images of gels. Boxes in *red* indicate selected Western blot results

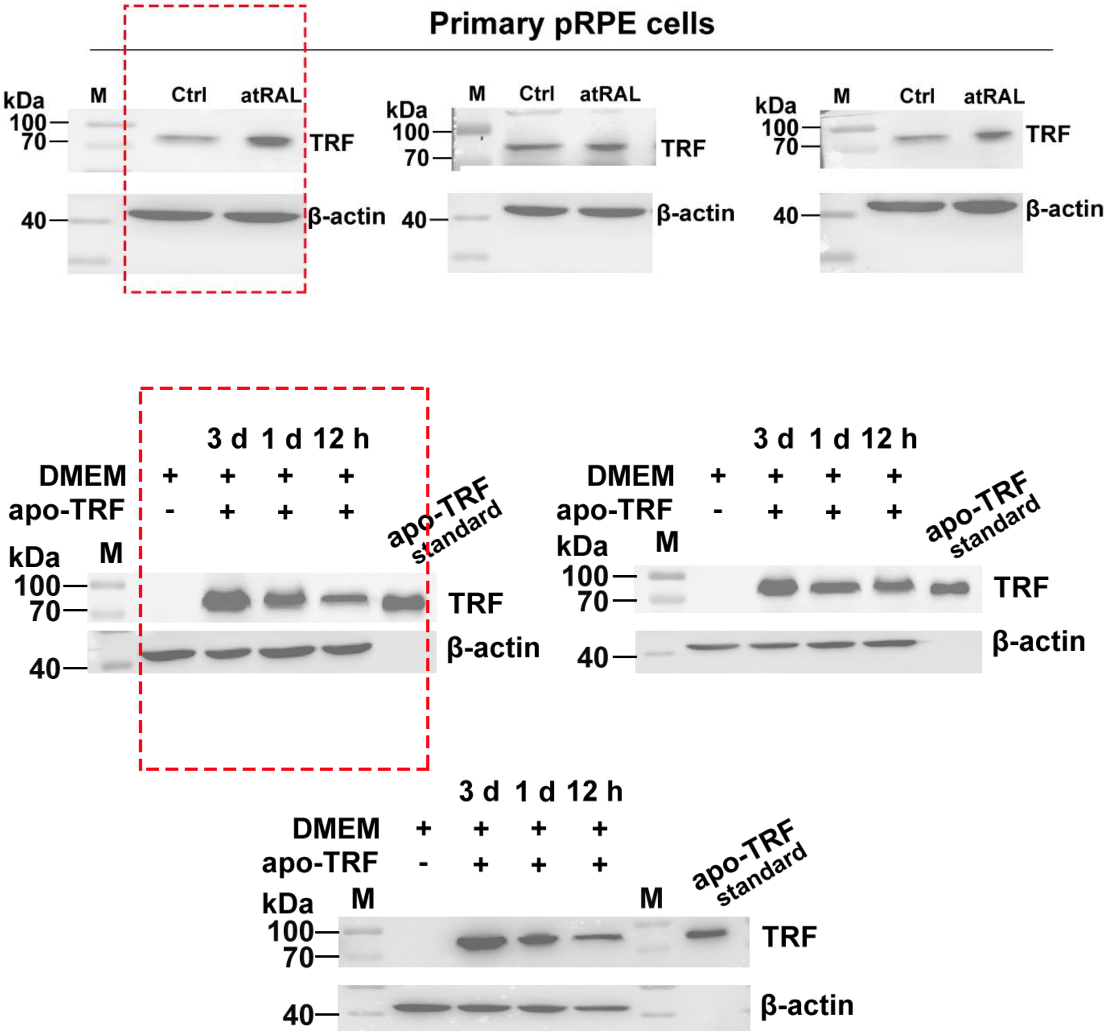

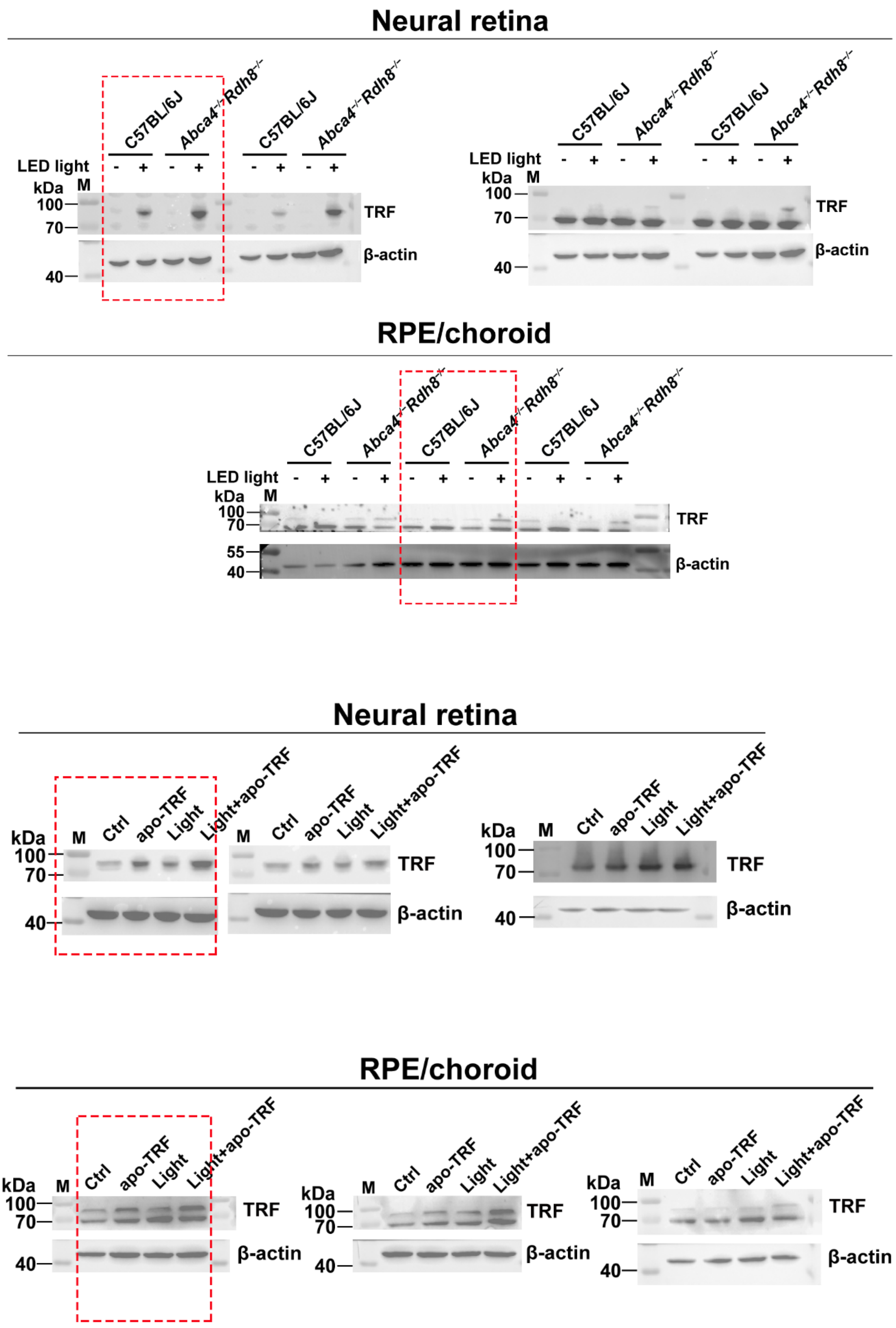
Neural retina
C57BL/6J
Abca4-/-Rdh8-/-
LED light
kDa
M
100
70
40
TRF
β-actin
RPE/choroid
55
Neural retina
Ctrl
apo-TRF
Light
Light+apo-TRF
RPE/choroid

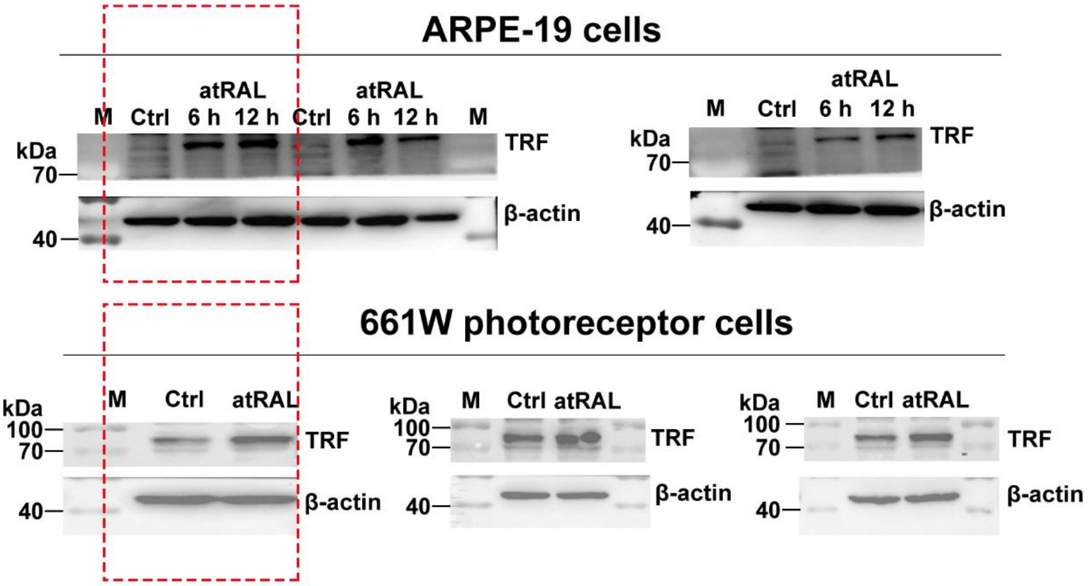
ARPE-19 cells
M Ctrl atRAL 6 h 12 h Ctrl atRAL 6 h 12 h M
kDa
70
40
TRF
β-actin
M Ctrl atRAL 6 h 12 h
kDa
70
40
TRF
β-actin
661W photoreceptor cells
kDa
100
70
40
M Ctrl atRAL
TRF
β-actin
kDa
100
70
40
M Ctrl atRAL
TRF
β-actin
kDa
100
70
40
M Ctrl atRAL
TRF
β-actin

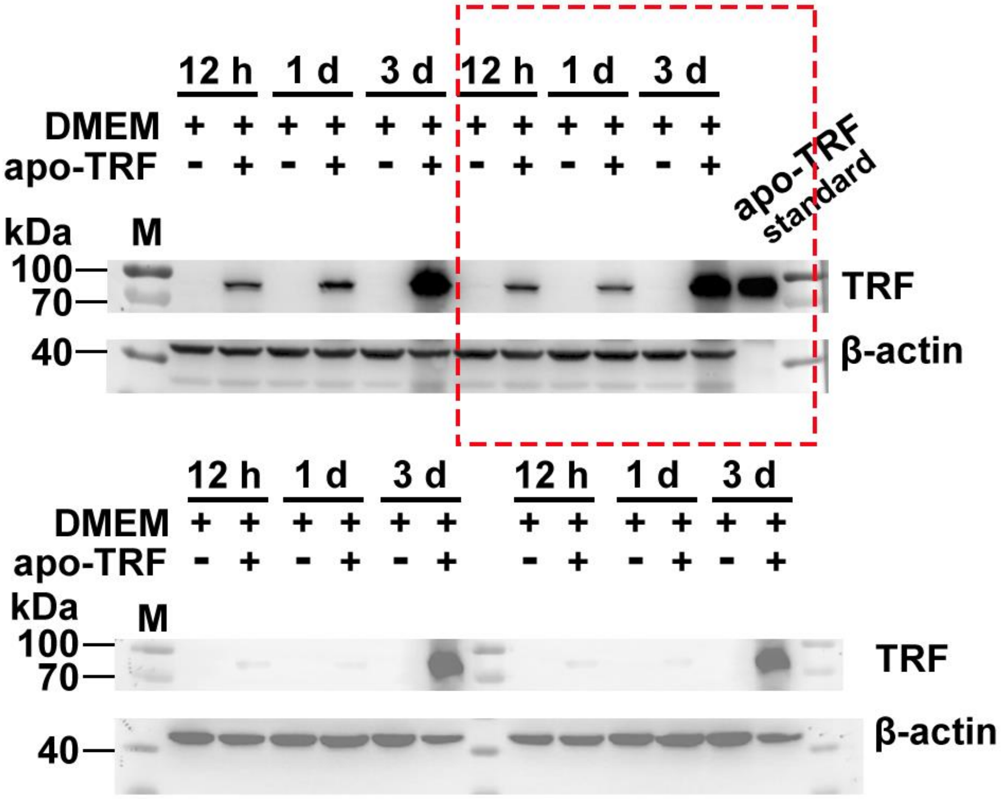
12 h 1 d 3 d 12 h 1 d 3 d
DMEM + + + + + + + + + + + +
apo-TRF - + - + - + - + - + - +
apo-TRF standard
kDa M
100
70
40
TRF
β-actin
12 h 1 d 3 d 12 h 1 d 3 d
DMEM + + + + + + + + + + + +
apo-TRF - + - + - + - + - + - +
kDa M
100
70
40
TRF
β-actin

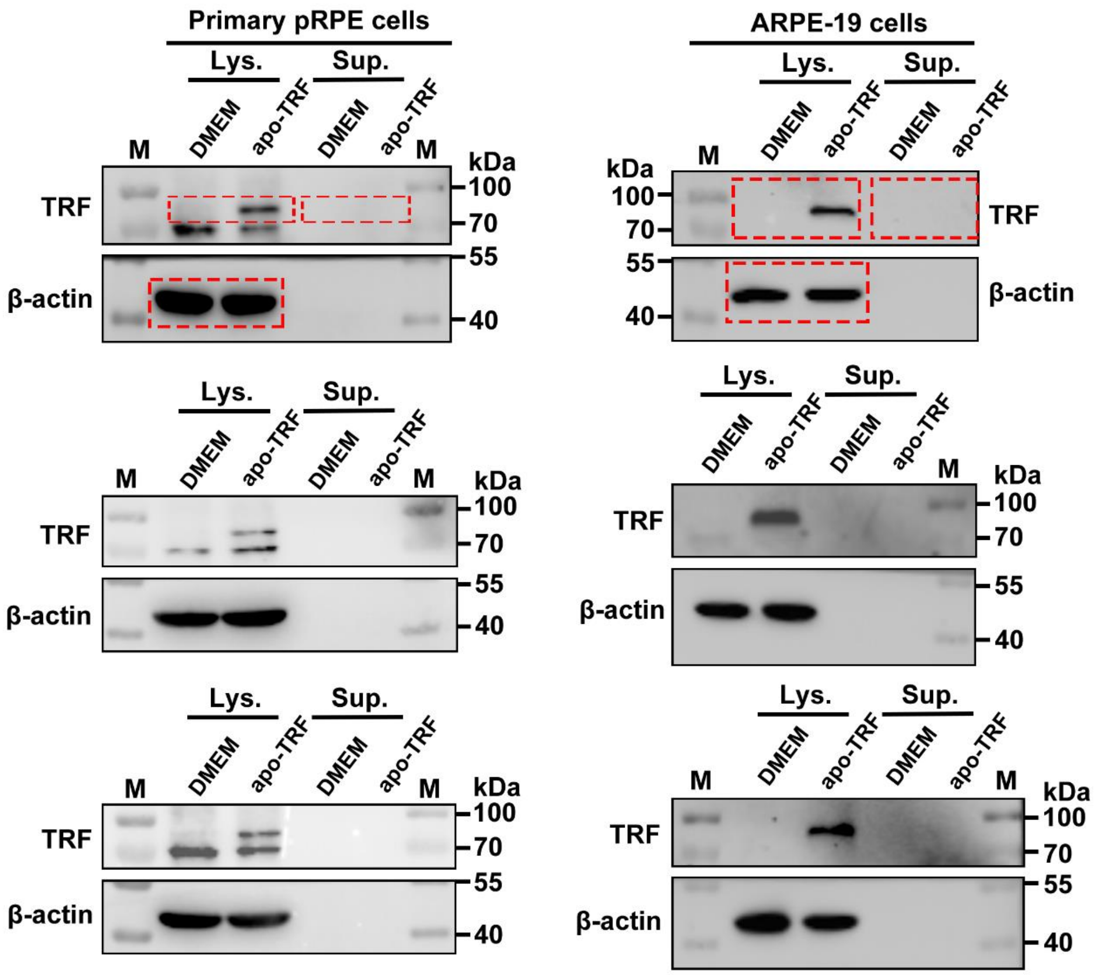

Primary pRPE cells
ARPE-19 cells
Lys.
Sup.
DMEM
apo-TRF
M
kDa
100
70
55
40
TRF
β-actin

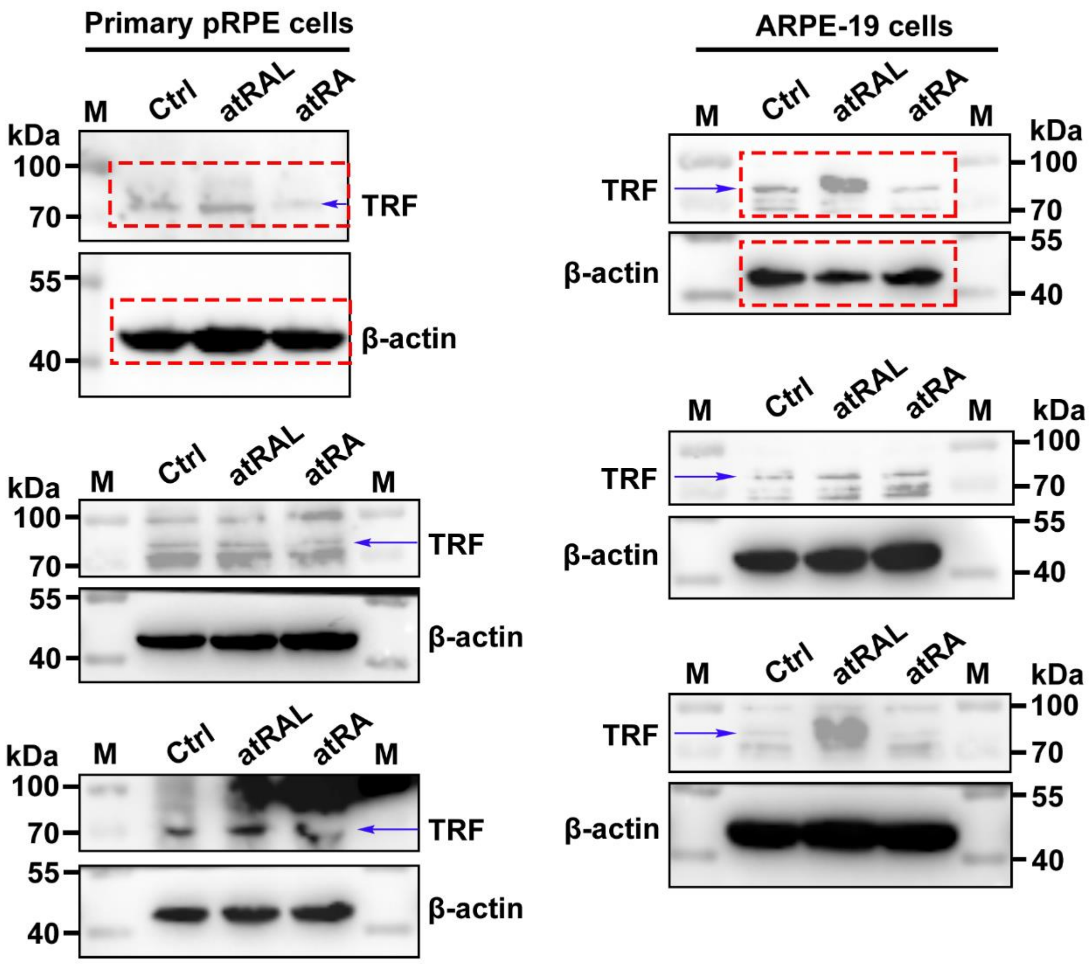
Primary pRPE cells
ARPE-19 cells
M
Ctrl
atRAL
atRA
kDa
100
70
55
40
TRF
β-actin
